# Term birth weight and ambient air pollutant concentrations during pregnancy, among women living in Monroe County, New York

**DOI:** 10.1038/s41370-019-0131-8

**Published:** 2019-04-02

**Authors:** Rui Li, Philip K. Hopke, Ann Dozier, Sally W. Thurston, Kelly Thevenet-Morrison, Daniel Croft, Mauro Masiol, Stefania Squizzato, David Chalupa, David Q. Rich

**Affiliations:** 1 Department of Public Health Sciences, University of Rochester Medical Center, Rochester, NY USA; 2 Department of Biostatistics and Computational Biology, University of Rochester Medical Center, Rochester, NY USA; 3 Department of Medicine, University of Rochester Medical Center, Rochester, NY USA; 4 Department of Environmental Medicine, University of Rochester Medical Center, Rochester, NY USA

**Keywords:** Ambient air pollution, Term birth weight, Epidemiology

## Abstract

Increased ambient air pollutant concentrations during pregnancy have been associated with reduced birth weight, but the etiologically relevant pregnancy time window(s) is/are unclear. In 76,500 singleton births in Monroe County, NY (2005–2016), who were 37–42 gestational weeks at delivery, we used generalized linear models to regress term birth weight against mean gestational month pollutant concentrations, adjusting for mean temperature, and maternal, infant, and medical service use characteristics. Overall, there were no clear patterns of term birth weight change associated with increased concentrations of any pollutant across gestational months. However, among Hispanic women only, increases in all pollutants, except O_3_, in multiple gestational months, were associated with decreased term birth weight. Each 3.25 µg/m^3^ increase in PM_2.5_ concentration in the 6^th^ gestational month was associated with a −20.4 g (95% CI = −34.0, −6.8) reduction in term birth weight among Hispanic women, but a 4.1 g (95% CI = −2.5, 10.8) increase among non-Hispanic mothers (*p* for interaction < 0.001). Although ambient air pollutant concentrations during pregnancy were not associated with reduced term birth weight among women of all ethnicities living in Monroe County, this observed association in Hispanic mothers may be a result of less exposure misclassification and bias (due to closer residential proximity to the monitoring site).

## Introduction

Birth weight is an important determinant of physical, psychological, and behavioral outcomes in later life [1] with some studies reporting associations between increased ambient air pollutant concentrations during pregnancy and reduced birth weight or an increased risk of low birth weight (LBW) (<2500 g) among term births (>37 weeks gestation at birth) [2–8], but not others [9]. In an international meta-analysis of 14 studies, increased mean concentrations of particulate air pollution across the entire pregnancy were associated with increased odds of low term birth weight (<2500 g) [5]. However, because this meta-analysis and these 14 studies did not identify specific gestational age windows (e.g., early or late pregnancy) during which exposure to air pollution was consistently associated with LBW, understanding potential mechanisms of any pollutant effect on fetal growth has been difficult [10]. In a study of pregnant Beijing women before, during, and after the 2008 Beijing Olympics, we found that infants of Beijing residents, whose 8^th^ month of gestation occurred during the 2008 Olympics and its large air pollution reductions, were heavier (23 g; 95% CI = 5, 40), on average, than infants whose 8^th^ month occurred during the same 2007 and 2009 calendar dates. Further, incremental increases in PM_2.5_, CO, SO_2_, and NO_2_ concentrations during the 8^th^ month of pregnancy were associated with 17–34 g decreases in term birth weight, respectively [9]. Although this study suggested late pregnancy air pollution exposure was important with regard to their effects on term birth weight and fetal growth, confirmation is needed in US cities with lower air pollution concentrations.

Increased risks of preterm birth [11] and preeclampsia, among women living in Monroe County, New York [12], have been associated with ambient concentrations of PM and gaseous pollutants during pregnancy, but associations with term birth weight have not been examined. Using a multi-year dataset of all births to women living in Monroe County and ambient pollutant concentrations measured at a central monitoring station in Rochester, NY, we hypothesized that increased mean pollutant concentrations in gestational months 7–9 (i.e., late pregnancy) would be associated with reduced term birth weight among term births. We then explored whether those associations were different by season, maternal employment, ethnicity, pregnancy complications, and infant gender.

## Material and methods

### Study population

We used maternal and infant data obtained from the Finger Lakes Region Perinatal Data System (FLRPDS), a collection of birth certificates and supplemental questionnaire data for births in the nine-county Finger Lakes region in New York State. Birth registrars at each hospital obtained data from mothers through interviews or surveys and abstracted data from mothers’ and infants’ medical (including prenatal) records. We included only singleton births to women residing in Monroe County at the time of birth from 1 January 2005 to 31 December 2016, with a gestational age of 37–42 completed weeks and a birth weight ≥ 500 g.

From the FLRPDS, we extracted data on infant and maternal characteristics, pregnancy complications, and medical service-related information. We also obtained information on the date of the last menstrual period (LMP). Gestational age was calculated primarily based on LMP, with clinical estimates used for those subjects missing the LMP date. We estimated the beginning and ending date of each gestational month (here, each month was 31 days), and used this to calculate mean pollutant concentrations for each gestational month. The mean pollutant concentrations for the last month was calculated for the last 31 days before the date of delivery. The primary outcome was term birth weight (in grams), but we also defined LBW as a term birth weight < 2500 g. The study was approved by the University of Rochester Medical Center Research Subjects Review Board.

### Air pollution and weather data

Hourly concentrations of air pollutants were collected from the New York State Department of Environmental Conservation (NYSDEC) site in Rochester. This site is adjacent to two major highways (I-490 and I-590) and State Route 96 on the east side of Rochester and measures hourly mass concentrations of fine particles (PM_2.5_, aerodynamic diameter < 2.5 µm), number concentrations of ultrafine particles (UFP, particles < 100 nm) and accumulation mode particles (AMP, particles with diameters of 100–470 nm), black carbon (BC, a marker of traffic pollution), sulfur dioxide (SO_2_), and ozone (O_3_). Temperature and relative humidity data measured at the Rochester International Airport were obtained from the National Oceanic and Atmospheric Administration (NOAA) National Climate Data Center. For each study subject’s gestational month of pregnancy, we calculated mean pollutant concentrations, temperature and relative humidity if at least 75% of the hourly pollutant, temperature, or humidity values during the specified gestational month period were reported. We then used these monthly pollutant concentrations and weather characteristics in the statistical analyses described below.

### Study design and statistical analyses

We used a cohort study design linking exposure to ambient pollutant concentrations during each gestational month with birth weight. We excluded births with an estimated date of conception before 17 April 2004 or after 12 March 2016 to avoid fixed cohort bias [13].

Using generalized linear models, we regressed each subject’s term birth weight (g) against their mean PM_2.5_ concentration in the 1^st^ gestational month, adjusting for gestational age (42, 41, 40, 39, 38, or 37 weeks), year of birth (2005–2016), sex of infant, month of conception (January–December), parity (1^st^ birth, 2^nd^ birth, or ≥ 3^rd^ birth), maternal education (less than high school, high school or graduate, some college or more), maternal country of birth (United States or non-United States), maternal race (White, Black, other, mixed), maternal ethnicity (non-Hispanic or Hispanic), maternal tobacco use during pregnancy (yes or no), maternal drug use during pregnancy (yes or no), pre-pregnancy body mass index (BMI) categories (underweight: <18.5 kg/m^2^, healthy weight: 18.5–24.9 kg/m^2^, overweight: 25.0–29.9 kg/m^2^, Class I obesity: 30.0–34.9 kg/m^2^, Class II obesity: 35.0–39.9 kg/m^2^, Class III obesity: ≥40 kg/m^2^), previous preterm birth (yes or no), previous cesarean section (yes or no), pre-pregnancy diabetes (yes or no), pre-pregnancy hypertension (yes or no), hospital of birth, trimester of first time prenatal visit (1^st^, 2^nd^, 3^rd^), primary provider of prenatal care (private physician, hospital outpatient, no provider), primary payer of prenatal care (Medicaid or family health plus or other government/child health plus B, private, self-pay, campus/Tricare or others), and mean temperature over the same gestational month. To determine what functional form of temperature should be included in the models, separate models were fit with temperature modeled either as a continuous variable (1 df) or using a natural spline with 2–4 degrees of freedom. A natural spline with 2 degrees of freedom had the lowest Akaike’s Information Criterion value and was thus included in the model. From this model, we estimated the difference in term birth weight (and 95% confidence interval) associated with each interquartile range (IQR) increase in mean PM_2.5_ concentration in the 1^st^ gestational month. We then reran this model for PM_2.5_ for each other gestational month (months 2, 3, 4, 5, 6, 7, 8, 9) as well as the last month of pregnancy (i.e., last 31 days of pregnancy), and other pollutants (BC, UFP, AMP, SO_2_, O_3_).

Next, we explored whether the association between air pollution and term birth weight varied by maternal ethnicity (non-Hispanic or Hispanic), by adding interaction terms between the mean pollutant concentration and Hispanic to our model. Similarly, we examined effect modification of the pollutant/term birth weight association by maternal employment during pregnancy (employed or not employed) as well as the presence of pregnancy complications (i.e., gestational diabetes and/or gestational hypertension and eclampsia) and/or adverse birth conditions (i.e., fetus at risk or abnormal birth conditions).

### Sensitivity analyses

Next, we separately added the mean O_3_ concentration from the same gestational month to each model with PM_2.5_, BC, UFP, AMP, or SO_2_ to estimate the difference in term birth weight associated with each IQR increase in pollutant concentration, independent of ozone. Second, using logistic regression with LBW as the outcome and the same set of covariates as in the main analyses, we estimated the odds of LBW associated with each IQR increase in each mean pollutant concentration during each gestational month. Data management and descriptive analyses were conducted using SAS v.9.4 (SAS Inc., Cary, NC, USA), and all the GLM and logistic analyses were conducted using R (version 2.15.0).

## Results

During the study period, there were 88,401 singleton live births in Monroe County compatible with the inclusion criteria. After excluding births with missing data for covariates included in our analytic models, we based our analyses of term birth weight on 76,500 subjects (86.5% of the total included subjects). Among the study subjects, the mean term birth weight was 3431.6 (±471.6) g and the overall percentage of term LBW infants was 2.15%. As shown in Table 1, LBW was more common among women who were younger, non-white, Hispanic, with less education, and among women who reported use of tobacco, alcohol, or drugs during pregnancy. LBW babies were also more common among women with lower pre-pregnancy BMI, with a previous cesarean section, gestational hypertension, preeclampsia, or eclampsia, and for infants of smaller gestational age, who were female, born in the winter, the first baby of the mother, and with the first prenatal visit happening during late pregnancy.

**Table 1:** Infant, maternal, and health service use characteristics of the study subjects in Monroe County, by birth weight groups, 2005–2016 (N = 76,500)

Variable	Low birth weight (<2500 g) (*n* = 1644, 2.1%)		Normal birth weight (≥25,000 g) (*n* = 74,856, 97.9%)	
	*n*	%	*n*	%
INFANT CHARACTERISTICS				
Gestational week				
37	675	41.1	4642	6.2
38	568	34.6	12,192	16.3
39	313	19.0	27,082	36.2
40	72	4.4	21,718	29.0
41	16	1.0	8839	11.8
42	0	0.0	383	0.5
Year of birth				
2005	115	7.0	5867	7.8
2006	123	7.5	6607	8.8
2007	112	6.8	6368	8.5
2008	138	8.4	6757	9.0
2009	154	9.4	6597	8.8
2010	155	9.4	6623	8.9
2011	153	9.3	6069	8.1
2012	137	8.3	5584	7.5
2013	145	8.8	6118	8.2
2014	142	8.6	6119	8.2
2015	147	8.9	6235	8.3
2016	123	7.5	5912	7.9
Sex of infant				
Male	659	40.1	38,174	51.0
Female	985	59.9	36,682	49.0
Season of conception				
Spring	403	24.5	17,236	23.0
Summer	403	24.5	19,548	26.1
Fall	423	25.7	19,872	26.6
Winter	415	25.2	18,200	24.3
Season of birth				
Spring	407	24.8	19,480	26.0
Summer	423	25.7	19,975	26.7
Fall	412	25.1	18,552	24.8
Winter	402	24.5	16,849	22.5
Month of conception				
January	136	8.3	6000	8.0
February	122	7.4	5652	7.6
March	121	7.4	5452	7.3
April	135	8.2	5497	7.3
May	147	8.9	6287	8.4
June	114	6.9	6357	8.5
July	131	8.0	6406	8.6
August	158	9.6	6785	9.1
September	142	8.6	6561	8.8
October	156	9.5	6724	9.0
November	125	7.6	6587	8.8
December	157	9.6	6548	8.8
Parity				
1^st^ birth	582	35.4	22,279	29.8
2^nd^ birth	377	22.9	21,444	28.7
3^rd^ birth or higher	685	41.7	31,133	41.6
Abnormal conditions of birth^a^	358	21.8	9831	13.1
Fetus at risk^b^	227	13.8	4544	6.1
MATERNAL CHARACTERISTICS				
Maternal age				
<18	60	3.7	1651	2.2
18–29	995	60.5	38,151	51.0
30–39	500	30.4	31,967	42.7
40+	52	3.2	2390	3.2
Unknown	37	2.3	697	0.9
Maternal education				
<High School	435	26.5	10,548	14.1
High school Graduate	461	28.0	16,414	21.9
Some college or more	748	45.5	47,894	64.0
Paternal education				
<High School	244	14.8	7336	9.8
High school Graduate	389	23.7	16,783	22.4
Some college or more	537	32.7	39,843	53.2
Unknown	474	28.8	10,894	14.6
Maternal country of birth				
US	1,484	90.3	66,797	89.2
Non-US	160	9.7	8059	10.8
Maternal employed during pregnancy	869	52.9	47,963	64.1
Maternal race				
White	770	46.8	52,376	70.0
Black	653	39.7	14,743	19.7
Other	166	10.1	6102	8.2
More than one	55	3.4	1635	2.2
Maternal ethnicity (non-Hispanic)	1,449	88.1	67,512	90.2
Maternal tobacco use				
No	1163	70.7	62,278	83.2
Yes	481	29.3	12,578	16.8
Maternal alcohol use				
No	1589	96.7	73,254	97.9
Yes	55	3.4	1583	2.1
Unknown	0	0.0	19	0.0
Maternal drug use				
No	1361	82.8	70,128	93.7
Yes	283	17.2	4728	6.3
Pre-pregnancy maternal BMI				
Underweight ( < 18.5 kg/m2)	125	7.6	2465	3.3
Healthy (18.5–24.9 kg/m2)	805	49.0	35,815	47.9
Overweight (25–29.9 kg/m2)	359	21.8	18,526	24.8
Class I Obesity (30–34.9 kg/m2)	181	11.0	9558	12.8
Class II Obesity (35–39.9 kg/m2)	117	7.1	4834	6.5
Class III Obesity ( ≥ 40 kg/m2)	57	3.5	3658	4.9
Previous preterm infant	135	8.2	2932	3.9
Previous cesarean section				
No	1434	87.2	63,776	85.2
Yes	210	12.8	11,080	14.8
Mom’s feeling toward this pregnancy				
Could be sooner	191	11.6	10,441	14.0
Just then	591	36.0	34,662	46.3
Could be later	481	29.3	16,556	22.1
Not want to be pregnant at all	150	9.1	4109	5.5
Diabetes: pre-pregnancy	17	1.0	595	0.8
Diabetes: gestational	68	4.1	4183	5.6
Hypertension: pre-pregnancy	75	4.6	1653	2.2
Gestational hypertension/preeclampsia	223	13.6	4485	6.0
Eclampsia	8	0.5	46	0.1
HEALTH SERVICE FACTORS				
Hospital at birth				
Hospital_A	472	28.7	25,253	33.7
Hospital_B	253	15.4	11,545	15.4
Hospital_C	395	24.0	18,617	24.9
Hospital_D	511	31.1	18,741	25.0
Hospital_Other	13	0.8	700	0.9
Trimester for first prenatal visit				
1st trimester	1122	68.3	59,244	79.1
2nd trimester	437	26.6	13,405	17.9
3rd trimester	85	5.2	2207	3.0
Primary provider of prenatal care				
Private physician	1096	66.7	61,572	82.3
Hospital outpatient	547	33.3	13,254	17.7
No provider	1	0.1	30	0.0
Primary payer for birth				
Medicaid, Family Health Plus, Child Health Plus	947	57.6	27,725	37.0
Private	669	40.7	45,962	61.4
Self-pay	17	1.0	462	0.6
Champus/Tricare	4	0.2	411	0.6
Others	7	0.4	296	0.4
Medicaid secondary payer				
No	1522	92.6	70,290	93.9
Yes	71	4.3	2466	3.3
Unknown	51	3.1	2100	2.8
Prenatal participation in WIC				
No	686	41.7	47,400	63.3
Yes	928	56.5	26,584	35.5
Unknown	30	1.8	872	1.2
Participation in HMO or other managed care plan				
No	774	47.1	41,970	56.1
Yes	834	50.7	31,245	41.7
Unknown	36	2.2	1641	2.2

^a^Abnormal conditions of birth: including assisted ventilation required immediately following delivery, assisted ventilation required for more than 6 h, NICU Admission (>4 h), newborn given surfactant replacement therapy, antibiotics received by the newborn for suspected neonatal sepsis, seizures or serious neurologic dysfunction, significant birth injury (Skeletal fx, peripheral nerve injury, soft tissue/solid organ hemorrhage) which requires intervention

^b^Fetus at Risk: reasons under indications for intervention (C-section, vacuum or forceps delivery)—In general, to mark “Fetus at Risk” there has to be an abnormal electronic fetal heart tracing and/or combined with an abnormal ultrasound: evidence from a biophysical profile of a disturbance in utero; positive contraction stress test, the presence of late decelerations, during oxytocin stimulation with half or more of the contractions; breech or a mal-presentation such as transverse lie, shoulder presentation; frank prolapse of the cord; fetal structural anomaly, such as fetal hydrocephalus; persistent late decelerations during most contractions; persistent variable decelerations during most contractions, often 60 to 80 bpm; prolonged bradycardia below 110 to 100 bpm 10 min or longer; prolonged tachycardia above 160 to 180 bpm persisting longer than 10 min; fetal scalp pH of less than 7.2. Includes acidosis

The mean and interquartile range of pollutant concentrations and weather variables were similar across each gestational month (Supplementary Table 1). Correlation coefficients for pollutant concentrations across gestational months and for pollutants within a gestational month are shown in Supplementary Table 2. Across gestational months, mean SO_2_ concentrations were highly correlated (0.781 > *r* < 0.902), while BC (0.251 > *r* < 0.737), UFP (0.469 > *r* < 0.758), and AMP (0.256 > *r* < 0.591) were moderately correlated. PM_2.5_ was only weakly correlated (0.035 > *r* < 0.503), while O3 was more variable, likely due to across-season patterns (−0.784 > *r* < 0.785). Within each gestational month, mean concentrations of PM_2.5_, BC, AMP, UFP, and SO_2_ were moderately correlated (e.g., Month 1: 0.431 > *r* < 0.719), while O_3_ was only minimally correlated with any other pollutant (e.g., Month 1: −0.162 > *r* < 0.161).

Inconsistent with our a priori hypothesis, increases in gestational month pollutant concentrations were generally not associated with decreased term birth weight among term births (Table 2). However, each IQR increase in BC concentration in the 1^st^ gestational month was associated with a 13.4 g increase in term birth weight (95% CI = 3.4, 23.4). Consistent with our a priori hypothesis, reduced term birth weight was associated with IQR increases in UFP concentrations in the 6^th^ gestational month (−7.5 g; 95% CI = −14.5, −0.4) and 7^th^ gestational month (−6.3 g, 95% CI = −13.3, 0.6), and increased O_3_ concentrations in the 1^st^ gestational month (−15.8 g, 95% CI = −31.5, −0.1). However, increased term birth weight was associated with IQR increases in mean O_3_ in the 7^th^ gestational month (17.2 g, 95% CI = 3.1, 31.4).

**Table 2:** Change in term birth weight (g) associated with each interquartile range (IQR) increase in mean gestational month pollutant concentrations

Pollutant	PM_2.5_ (IQR = 3.25 µg/m^3^)		BC (IQR = 0.28 µg/m^3^)		UFP (IQR = 1800 N/cm^3^)		AMP (IQR = 400 N/cm^3^)		SO_2_ (IQR = 2.65 ppb)		O_3_ (IQR = 0.012 ppb)	
Gestational month	*n*	Birth weight change (g) (95% CI)	*n*	Birth weight change (g) (95% CI)	*n*	Birth weight change (g) (95% CI)	*n*	Birth weight change (g) (95% CI)	*n*	Birth weight change (g) (95% CI)	*n*	Birth weight change (g) (95% CI)
1	68,173	−2.1 (−9.2, 4.9)	68,949	13.4 (3.4, 23.4)	63,712	5.5 (−1.6, 12.7)	62,193	5.4 (−2.7, 13.5)	74,198	−1.8 (−17.3, 13.7)	74,326	−15.8 (−31.5, −0.1)
2	68,788	1.1 (−5.9, 8.1)	69,590	−3.0 (−12.8, 6.9)	64,173	0.8 (−6.5, 8.1)	62,651	−4.9 (−13.0, 3.2)	74,751	2.3 (−13.0, 17.6)	74,751	1.9 (−13.8, 17.6)
3	69,152	−1.3 (−8.2, 5.6)	70,070	−1.6 (−11.2, 7.9)	64,808	−0.6 (−8.1, 6.9)	63,300	−0.8 (−8.9, 7.3)	74,751	−2.2 (−17.5, 13.1)	74,751	10.0 (−5.6, 25.6)
4	69,830	2.4 (−4.4, 9.1)	70,483	−1.6 (−11.0, 7.7)	65,142	1.0 (−6.5, 8.6)	63,681	0.1 (−7.9, 8.1)	74,558	−2.6 (−17.9, 12.7)	74,558	11.0 (−4.3, 26.2)
5	70,335	−1.6 (−8.3, 5.1)	71,023	2.1 (−7.3, 11.5)	65,739	−1.3 (−8.7, 6.0)	64,348	2.0 (−6.1, 10.1)	74,425	−0.6 (−16.1, 14.9)	74,425	−7.6 (−22.3, 7.2)
6	71,019	1.9 (−4.7, 8.5)	71,567	5.1 (−4.4, 14.5)	65,972	−7.5 (−14.5, −0.4)	64,563	−0.1 (−7.9, 7.7)	74,381	2.1 (−13.4, 17.6)	74,381	0.9 (−13.6, 15.3)
7	71,668	0.6 (−5.3, 6.8)	72,079	−5.8 (−15.3, 3.8)	66,422	−6.3 (−13.3, 0.6)	64,996	−5.2 (−13.1, 2.6)	74,470	−3.2 (−19.1, 12.7)	74,470	17.2 (3.1, 31.4)
8	72,209	0.7 (−5.5, 6.9)	72,479	−0.0 (−9.6, 9.6)	66,825	4.4 (−2.4, 11.2)	65,409	2.6 (−5.2, 10.5)	74,342	−0.4 (−16.9, 16.1)	74,342	−0.9 (−15.1, 13.3)
9	72,694	2.4 (−3.8, 8.5)	73,009	−6.1 (−15.9, 3.6)	66,972	1.1 (−5.7, 7.9)	65,503	−2.8 (−10.6, 5.0)	74,297	−9.1 (−25.4, 7.2)	74,297	0.5 (−13.8, 14.9)
Last 31 days	72,661	−3.9 (−9.9, 2.1)	72,885	−6.0 (−15.5, 3.6)	66,968	−1.5 (−8.1, 5.1)	65,544	−3.4 (−10.9, 4.2)	74,281	−11.1 (−27.0, 4.8)	74,281	0.6 (−12.1, 13.2)

Models adjusted for temperature of each gestational month, gestational ages, year of birth, month of conception, sex of infant, parity, maternal education, maternal country of birth, maternal race, maternal ethnicity, maternal tobacco use, maternal drug use, maternal pre-pregnancy BMI, previous preterm birth, previous cesarean section, pre-pregnancy diabetes, pre-pregnancy hypertension, hospital of birth, trimester of first prenatal care visit, primary provider of prenatal care, primary payer for prenatal care

*PM*_2.5_: fine particles (aerodynamic diameter < 2.5 µm). *UFP* ultrafine particles (particles with diameters < 100 nm), *AMP* accumulation mode particles (particles with diameters 100–470 nm), *BC* black carbon (a marker of traffic pollution), *SO*_*2*_ sulfur dioxide, *O*_*3*_ *ozone*

There were also no clear patterns of effect modification of the air pollutant/term birth weight associations by infant gender (Table 3; Supplementary Table 3). However, among male infants, each IQR increase in PM_2.5_ concentration in the 3^rd^ gestational month was associated with a 5.6 g decrease in term birth weight (95% CI = −13.5, 2.3), while among females it was associated with a 3.1 g increase (95% CI = −4.8, 11.0; *p*-value for interaction = 0.024) (Table 3). Although there was no clear pattern of effect modification by pregnancy complications (Table 3, Supplementary Table 4), among pregnancies with complications each IQR increase in PM_2.5_ concentration in the 6^th^ gestational month was associated with a significantly (*p* = 0.010) greater increase in term birth weight (10.3 g; 95% CI = 1.1, 19.5) than in those pregnancies without complications (−1.0 g; 95% CI = −7.9, 5.9) (Table 4). Among pregnancies where the mother was unemployed, decreases in term birth weight associated with IQR increases in PM_2.5_ concentration in the last 31 days (−9.6 g; 95% CI = −17.3, −1.8) were significantly (*p* = 0.025) larger in magnitude than among employed mothers (−0.9 g; 95% CI = −7.4, 5.7) (Table 3). IQR increases in PM_2.5_ concentration in the 1^st^ and 9^th^ months followed similar patterns, but there was no pattern of effect modification of associations between other pollutants and term birth weight (Supplementary Table 5). However, there were clear differences in term birth weight changes associated with IQR increases in mean PM_2.5_ concentrations in multiple gestational months for Hispanic versus non-Hispanic women (e.g., Month 1—non-Hispanic: 0.6 g, 95% CI = −7.8, 6.6; Hispanic: −16.5 g, 95% CI = −30.3, −2.7; *p* = 0.017; Table 4). Similar patterns of effect modification by ethnicity were observed for all other pollutants at most gestational months, except O_3_ (Supplementary Table 6).

**Table 3:** Term birth weight change (g) associated with each interquartile range (IQR) increase in mean PM_2.5_ concentrations during each gestational month, by infant gender, maternal ethnicity, maternal employment, and pregnant complications

Gestational month	INFANT GENDER					MATERNAL ETHNICITY				
	Male		Female		*p* for interaction	Hispanic		Non-Hispanic		*p* for interaction
	*n*	Birth weight change (g) (95% CI)	*n*	Birth weight change (g) (95% CI)		*n*	Birth weight Change (g) (95% CI)	*n*	Birth weight change (g) (95% CI)	
1	34,558	0.8 (−7.2, 8.8)	33,615	−5.2 (−13.2, 2.9)	0.126	6787	−16.5 (−30.3, −2.7)	61,386	−0.6 (−7.8, 6.6)	0.017
2	34,855	−0.1 (−8.1, 7.8)	33,933	2.3 (−5.6, 10.3)	0.526	6847	−10.5 (−24.3, 3.3)	61,941	2.3 (−4.8, 9.4)	0.056
3	35,007	−5.6 (−13.5, 2.3)	34,145	3.1 (−4.8, 11.0)	0.024	6885	−8.9 (−22.7, 4.9)	62,267	−0.5 (−7.5, 6.5)	0.209
4	35,397	−0.4 (−8.1, 7.3)	34,433	5.3 (−2.5, 13.1)	0.145	6918	−5.6 (−19.1, 8.0)	62,912	3.2 (−3.7, 10.0)	0.186
5	35,650	−3.0 (−10.7, 4.7)	34,685	−0.2 (−7.9, 7.5)	0.467	6996	−15.8 (−29.3, −2.3)	63,339	−0.1 (−6.9, 6.7)	0.018
6	36,031	3.5 (−4.0, 11.1)	34,988	0.2 (−7.4, 7.9)	0.395	7040	−20.4 (−34.0, −6.8)	63,979	4.1 (−2.5, 10.8)	<0.001
7	36,371	1.9 (−5.3, 9.1)	35,297	−0.7 (−7.9, 6.5)	0.497	7102	−10.8 (−23.8, 2.3)	64,566	1.8 (−4.5, 8.1)	0.052
8	36,639	1.1 (−6.1, 8.3)	35,570	0.4 (−6.9, 7.6)	0.851	7170	−10.2 (−23.1, 2.6)	65,039	1.9 (−4.4, 8.3)	0.057
9	36,842	−0.6 (−7.7, 6.6)	35,852	5.4 (−1.8, 12.5)	0.109	7194	−6.0 (−18.9, 7.0)	65,500	3.2 (−3.0, 9.5)	0.151
Last 31 days	36,851	−6.8 (−13.8, 0.2)	35,810	−1.0 (−8.0, 6.1)	0.117	7177	−16.4 (−29.1, −3.6)	65,484	−2.6 (−8.1, 3.5)	0.030

Gestational month	MATERNAL EMPLOYMENT					PREGNANCY COMPLICATIONS ^a^				
	Employed		Unemployed		*p* for interaction	No		Yes		*p* for interaction
	*n*	Birth weight change (g) (95% CI)	*n*	Birth weight change (g) (95% CI)		*n*	Birth weight change (g) (95% CI)	*n*	Birth weight change (g) (95% CI)	
1	43,607	0.7 (−6.9, 8.3)	24,566	−7.2 (−16.0, 1.5)	0.051	50,332	−2.4 (−9.8, 5.0)	17,841	−1.3 (−10.9, 8.3)	0.812
2	43,978	3.4 (−4.2, 11.0)	24,810	−3.0 (−11.6, 5.7)	0.117	50,824	0.9 (−6.4, 8.3)	17,964	1.5 (−8.0, 11.0)	0.891
3	44,153	0.3 (−7.2, 7.8)	24,999	−4.0 (−12.6, 4.6)	0.293	51,119	−1.5 (−8.7, 5.8)	18,033	−1.0 (−10.5, 8.4)	0.919
4	44,605	3.0 (−4.4, 10.3)	25,225	1.3 (−7.1, 9.7)	0.683	51,634	1.5 (−5.6, 8.6)	18,196	4.4 (−4.9, 13.7)	0.504
5	44,875	−2.9 (−10.2, 4.4)	25,460	0.5 (−7.9, 8.9)	0.399	51,957	−2.2 (−9.3, 4.8)	18,378	0.4 (−8.9, 9.7)	0.554
6	45,351	2.0 (−5.2, 9.1)	25,668	1.8 (−6.5, 10.1)	0.968	52,460	−1.0 (−7.9, 5.9)	18,559	10.3 (1.1, 19.5)	0.010
7	45,763	1.1 (−5.7, 7.8)	25,905	−0.2 (−8.1, 7.8)	0.749	52,907	−0.7 (−7.3, 5.8)	18,761	4.6 (−4.2, 13.3)	0.219
8	46,087	2.5 (−4.3, 9.3)	26,122	−2.6 (−10.5, 5.4)	0.193	53,327	0.7 (−5.9, 7.3)	18,882	0.8 (−7.9, 9.5)	0.983
9	46,403	5.3 (−1.4, 12.0)	26,291	−3.0 (−10.9, 4.9)	0.031	53,711	2.5 (−4.0, 9.1)	18,983	2.0 (−6.7, 10.6)	0.895
Last 31 days	43,607	−0.9 (−7.4, 5.7)	24,566	−9.6 (−17.3, −1.8)	0.025	53,668	−3.8 (−10.1, 2.6)	18,993	−4.3 (−12.8, 4.3)	0.908

^a^Including gestational diabetes, gestational hypertension, eclampsia, abnormal birth conditions, and fetus at risk

**Table 4:** Relative odds of low birth weight (LBW) associated with each interquartile range (IQR) increase in mean pollutant concentration, by gestational month

Pollutant	PM_2.5_ (IQR = 3.25 µg/m^3^)		BC (IQR = 0.28 µg/m^3^)		UFP (IQR = 1800 N/cm^3^)		AMP (IQR = 400 N/cm^3^)		SO_2_ (IQR = 2.65 ppb)		O_3_ (IQR = 0.012 ppb)	
Gestational months	*n*	Odds ratio (95% CI)	*n*	Odds ratio (95% CI)	*n*	Odds ratio (95% CI)	*n*	Odds ratio (95% CI)	*n*	Odds ratio (95% CI)	*n*	Odds ratio (95% CI)
1	68,173	1.03 (0.91, 1.16)	68,949	0.98 (0.82, 1.16)	63,712	1.00 (0.88, 1.13)	62,193	1.14 (0.98, 1.31)	74,198	1.05 (0.79, 1.38)	74,326	1.22 (0.92, 1.62)
2	68,788	0.98 (0.87, 1.12)	69,590	1.06 (0.90, 1.26)	64,173	0.95 (0.84, 1.09)	62,651	0.94 (0.82, 1.09)	74,751	0.79 (0.61, 1.05)	74,751	1.03 (0.78, 1.36)
3	69,152	1.03 (0.90, 1.16)	70,070	1.08 (0.92, 1.28)	64,808	1.00 (0.88, 1.15)	63,300	1.03 (0.90, 1.19)	74,751	0.90 (0.68, 1.19)	74,751	1.07 (0.82, 1.42)
4	69,830	1.00 (0.88, 1.13)	70,483	0.99 (0.84, 1.17)	65,142	0.89 (0.77, 1.01)	63,681	0.92 (0.80, 1.06)	74,558	0.96 (0.73, 1.27)	74,558	1.11 (0.84, 1.45)
5	70,335	0.97 (0.86, 1.09)	71,023	1.07 (0.90, 1.27)	65,739	1.01 (0.88, 1.15)	64,348	1.03 (0.89, 1.20)	74,425	0.93 (0.70, 1.25)	74,425	0.99 (0.76, 1.28)
6	71,019	1.06 (0.95, 1.20)	71,567	1.04 (0.89, 1.23)	65,972	1.06 (0.93, 1.21)	64,563	1.07 (0.93, 1.23)	74,381	1.30 (0.98, 1.72)	74,381	1.01 (0.79, 1.30)
7	71,668	0.90 (0.80, 1.00)	72,079	0.93 (0.79, 1.11)	66,422	1.08 (0.96, 1.23)	64,996	0.98 (0.85, 1.14)	74,470	0.90 (0.67, 1.21)	74,470	0.78 (0.61, 1.00)
8	72,209	0.98 (0.88, 1.09)	72,479	0.95 (0.80, 1.14)	66,825	0.90 (0.79, 1.00)	65,409	0.91 (0.80, 1.05)	74,342	0.85 (0.63, 1.15)	74,342	0.77 (0.59, 0.99)
9	72,694	1.00 (0.90, 1.11)	73,009	1.01 (0.84, 1.20)	66,972	1.03 (0.91, 1.16)	65,503	0.98 (0.85, 1.13)	74,297	1.01 (0.75, 1.35)	74,297	0.86 (0.66, 1.12)
Last 31 days	72,661	1.07 (0.96, 1.19)	68,949	0.98 (0.82, 1.16)	64,173	0.95 (0.84, 1.09)	63,300	1.03 (0.90, 1.19)	74,281	0.98 (0.73, 1.31)	74,297	0.86 (0.66, 1.12)

Models adjusted for temperature of each gestational month, gestational ages, year of birth, month of conception, sex of infant, parity, maternal education, maternal country of birth, maternal race, maternal ethnicity, maternal tobacco use, maternal drug use, maternal pre-pregnancy BMI, previous preterm birth, previous cesarean section, pre-pregnancy diabetes, pre-pregnancy hypertension, hospital of birth, trimester of first prenatal care visit, primary provider of prenatal care, primary payer for prenatal care

*PM*_2.5_: fine particles (aerodynamic diameter < 2.5 µm). *UFP* ultrafine particles (particles with diameters < 100 nm), *AMP* accumulation mode particles (particles with diameters 100–470 nm), *BC* black carbon (a marker of traffic pollution), *SO*_*2*_ sulfur dioxide, O_*3*_ ozone

When adjusting for the mean O_3_ concentration in the same gestational month, we did not observe any associations between IQR increases in pollutant concentrations and term birth weight, except for BC during the 1^st^ gestational month (IQR = 0.28 µg/m^3^; 11.6 g, 95% CI = 1.5, 21.7) (Supplementary Table 7). Similar to our main analysis findings with term birth weight as the outcome, we did not find any increased odds of LBW associated with IQR increases in concentrations of any pollutant during any gestational month (Table 4).

## Discussion

Using a large dataset combining birth certificates and maternal and infant hospital discharge data in the Finger Lakes region of New York State and 12 years of air pollution monitoring data, we did not find consistent associations between term birth weight and concentrations of any pollutant (i.e., PM_2.5_, BC, UFP, AMP, SO_2_, and O_3_) during any gestational month, after adjustment for numerous maternal, infant and birth characteristics, and temporal factors (i.e., season and long term time trend). Results were similar after additional adjustment for ambient O_3_ concentrations, as well as when estimating pollutant effects on the odds of LBW. There were no differences in term birth weight changes associated with air pollutants by maternal employment during pregnancy, pregnancy complications, adverse birth conditions, or infant gender. However, infants born to unemployed mothers had slightly larger reductions in term birth weight associated with increased pollutant concentrations than employed mothers. Further, across all gestational months and multiple pollutants, we observed large decreases in term birth weight associated with increased gestational month pollutant concentrations among Hispanic mothers, but not among non-Hispanic mothers.

Our finding of no association between air pollution levels during pregnancy and term birth weight among term birth is inconsistent with most previous studies. A pooled analysis of 14 European birth cohort studies reported a −7 g reduction in term birth weight (95% CI = −17, 2) associated with each 5 µg/m³ increase in PM_2.5_ concentration across the entire pregnancy [4]. An increased risk of LBW associated with increased gestational PM pollutant concentrations was reported in a meta-analyses of multi-continent studies [5]. In the United States, an increased risk of LBW was associated with PM_2.5_ concentrations in both early and late pregnancy (1^st^ and 3^rd^ trimesters in a New Jersey study [14]. In Massachusetts and Connecticut, each 2.2 µg/m^3^ increase in PM_2.5_ concentrations during pregnancy was associated with a –14.7 g reduction (–17.1 to –12.3) in birth weight among term births (37–42 gestational weeks) [15]. In Beijing, interquartile range increases in air pollutant concentrations during the 8^th^ month were associated with 17 g to 34 g reductions in term birth weight among term births. In the same study, pregnancies with their 8^th^ gestational months during the 2008 Beijing Olympics (and its large declines in air pollutant concentrations during the Games) were 23 g larger (95% CI = 5, 40) than pregnancies with their 8^th^ months of pregnancy during the same calendar dates in 2007 or 2009 [9]. However, other studies reported no such associations [7].

With regards to other studies of air pollution and birth outcomes in residents of New York State, our findings are consistent with those of Brown et al. [16], who found no association between exposure to PM_2.5_ during pregnancy and term LBW. Previously, in Rochester NY, Pereira et al. reported that elevated ambient PM_2.5_ levels during pregnancy were associated with an increased odds of preterm birth, but not pre-labor rupture of membranes [11]. In another study examining the association between ambient pollutants and hypertensive disorder of pregnancy in New York City, no association was found between PM_2.5_ or NO_2_ concentrations and gestational hypertension [17]. Thus, the role of ambient air pollution exposure during pregnancy in New York State on birth outcomes remains unclear.

Our findings of no effect modification by infant gender is consistent with some studies [8, 18], but not another [19] who reported that each ~30 µg/m^3^ increase in PM_2.5_ concentration during gestation was associated with a 189 g deficit in male newborns, but only a 17 g deficit in female newborns.[19] In a systematic review, Ghosh et al. [20] reported a higher prevalence of LBW at birth among female than male infants, but a higher risk of LBW associated with pollutant concentrations among male infants.

**Fig. 1:**
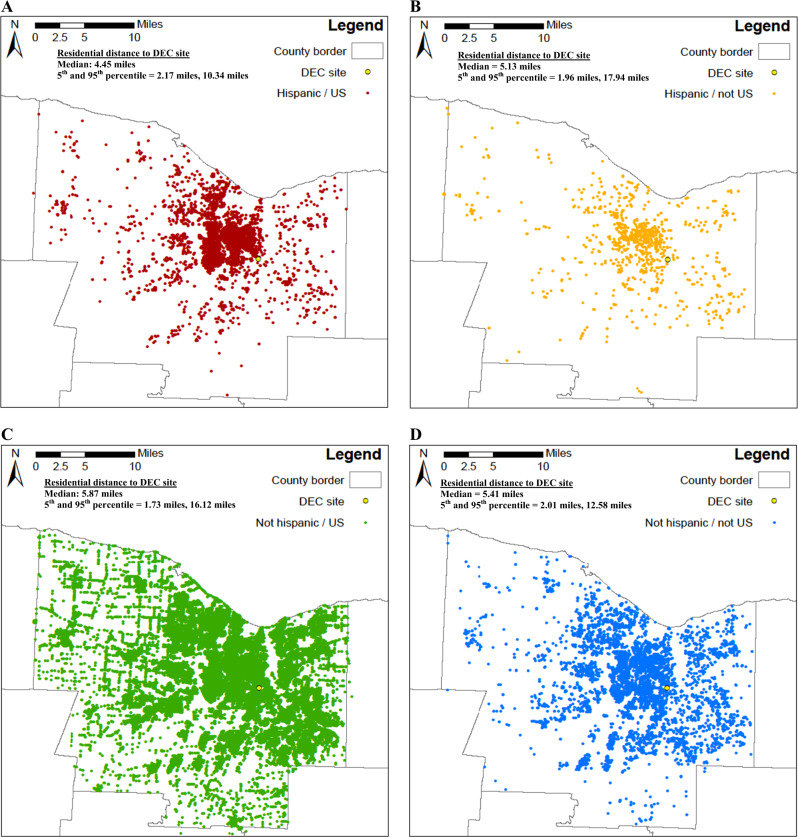
Residence of each study subject, separately for: **a** Hispanic mothers who were born in the United States. **b** Hispanic mothers who were not born in the United States. **c** Non-Hispanic mothers who were born in the United States. **d** Non-Hispanic mothers who were not born in the United States

Our finding of decreased term birth weight associated with increased concentrations of multiple pollutants among Hispanic mothers, but not non-Hispanic mothers, may be due to several factors. Some have reported that mothers with lower socio-economic status are more vulnerable to air pollution exposure during pregnancy due in part to having residences in areas of high air pollution, and/or longer times spent commuting and thus higher traffic pollution exposures [4, 21]. Further, they argue that these mothers are also more likely to have lower birth weight and a higher risk of LBW [4, 21]. Another explanation might be different degrees of exposure misclassification for Hispanic and non-Hispanic mothers. Using GIS, we mapped the residence of each mother in our study, and found that Hispanic mothers, both those born in the US (median residential distance from DEC site = 4.45 miles) and not born in the United States (median distance = 5.13 miles) generally lived closer to the monitoring station than non-Hispanic mothers born in the US (median distance = 5.87 miles) and non-Hispanic mothers not born in the United States (median distance = 5.41 miles; Fig. 1). Thus, the PM_2.5_ concentrations from the monitoring location may be better estimates of Hispanic mothers’ exposures to ambient PM_2.5_ during pregnancy than non-Hispanic mothers (i.e., less exposure error), resulting in less underestimation of effect estimates for Hispanic mothers than non-Hispanic mothers. Thus, this may, in part, explain our findings.

Although our study had several strengths including a large sample size with resulting increased statistical power, and the use of a detailed database of linked birth certificates and hospital admissions data, there are several limitations that should be considered when making inference. First, we used pollution data from the single central monitoring site as the proxy for each pregnant woman’s individual exposure to air pollution during pregnancy, but study subjects lived a median of 5.65 miles from the monitoring station (5^th^ percentile = 1.80 miles, 95^th^ percentile = 15.67 miles). Thus, this likely resulted in non-differential exposure misclassification and underestimates of the true effects. Our previous work has suggested spatial heterogeneity in the PM_2.5_ concentrations across Rochester [22]. However, other pollutants may be more spatially heterogeneous [23–26]. Further, there are other modifiers of air pollution exposure (e.g., time spent indoors versus outdoors) that all could have resulted in non-differential exposure misclassification and effect underestimation. Second, tobacco use, alcohol consumption and drug use were collected through self-report and therefore may be underreported. Although this may lead to residual confounding by these factors, there were other SES covariates included in the analysis (some of which are likely correlated with tobacco, alcohol, and drug use). Thus, residual confounding by these factors is likely minimal. Third, although we ran many models, our inference was based largely on the overall pattern of term birth weight changes associated with increased pollution concentrations across gestational months, and not on whether each individual effect estimate was statistically significant.

In summary, among pregnant women of Monroe County, New York from 2005 to 2016, we found no clear patterns of term birth weight change associated with increased concentrations of any pollutant across gestational months. Further, there were no patterns of effect modification by infant gender, pregnancy complications, or season. However, among Hispanic women only, increases in all pollutants, except O_3_, in multiple gestational months, were associated with decreased term birth weight. This may be a result of different degrees of exposure misclassification due to residential proximity to the air pollutant monitoring site, but further work is needed to understand these differences by ethnicity.

## Supplementary information

Supplementary Tables
