# Supplementary material for: Term birth weight and ambient air pollutant concentrations during pregnancy, among women living in Monroe County, New York
Source: J Expo Sci Environ Epidemiol. 2019 Apr 2;29(4):500–9. doi: 10.1038/s41370-019-0131-8 (PMC6592743; doi:10.1038/s41370-019-0131-8)
Supplement: Supplementary file 1 — Supplementary Tables [file 41370_2019_131_MOESM1_ESM.docx]

**Supplementary Table 1.** Descriptive statistics of mean gestational month air pollutant concentrations, temperature, and relative humidity for study subjects (2005-2016).

| **Pollutant** | **Month of Gestation** | **n** | **Mean** | **Standard Deviation** | **Min** | **5^th^** | **25^th^** | **50^th^** | **75^th^** | **95^th^** | **Max** | **IQR** |
| --- | --- | --- | --- | --- | --- | --- | --- | --- | --- | --- | --- | --- |
| **PM_2.5_**  **(µg/m^3^)** | 1 | 78,443 | 8.74 | 2.62 | 2.97 | 5.07 | 6.93 | 8.33 | 10.21 | 13.87 | 18.75 | 3.28 |
|  | 2 | 79,131 | 8.73 | 2.63 | 2.97 | 5.08 | 6.92 | 8.30 | 10.20 | 13.85 | 18.75 | 3.28 |
|  | 3 | 79,441 | 8.69 | 2.61 | 2.97 | 5.07 | 6.89 | 8.28 | 10.16 | 13.82 | 18.75 | 3.27 |
|  | 4 | 80,290 | 8.67 | 2.60 | 2.97 | 5.08 | 6.87 | 8.26 | 10.14 | 13.78 | 18.75 | 3.27 |
|  | 5 | 80,785 | 8.65 | 2.59 | 2.97 | 5.09 | 6.87 | 8.21 | 10.10 | 13.76 | 18.75 | 3.23 |
|  | 6 | 81,523 | 8.66 | 2.59 | 2.97 | 5.10 | 6.88 | 8.22 | 10.13 | 13.75 | 18.75 | 3.25 |
|  | 7 | 82,291 | 8.70 | 2.64 | 2.97 | 5.10 | 6.89 | 8.25 | 10.16 | 13.85 | 18.75 | 3.27 |
|  | 8 | 82,849 | 8.68 | 2.64 | 2.97 | 5.03 | 6.88 | 8.24 | 10.18 | 13.84 | 18.75 | 3.30 |
|  | 9 | 83,406 | 8.67 | 2.67 | 2.97 | 5.01 | 6.85 | 8.20 | 10.13 | 14.01 | 18.75 | 3.28 |
|  | Last 31 days | 83,393 | 8.67 | 2.67 | 2.97 | 5.01 | 6.85 | 8.20 | 10.13 | 13.87 | 18.75 | 3.28 |
| **UFP**  **(N/cm^3^)** | 1 | 73,275 | 4410 | 1650 | 114 | 2129 | 3345 | 4197 | 5237 | 7601 | 10545 | 1892 |
|  | 2 | 73,712 | 4395 | 1639 | 114 | 2133 | 3343 | 4178 | 5229 | 7579 | 10545 | 1886 |
|  | 3 | 74,410 | 4406 | 1645 | 114 | 2139 | 3345 | 4189 | 5234 | 7601 | 10545 | 1889 |
|  | 4 | 74,809 | 4400 | 1636 | 114 | 2139 | 3351 | 4181 | 5201 | 7591 | 10545 | 1849 |
|  | 5 | 75,474 | 4406 | 1634 | 114 | 2152 | 3354 | 4194 | 5198 | 7605 | 10545 | 1844 |
|  | 6 | 75,770 | 4389 | 1624 | 114 | 2144 | 3350 | 4174 | 5170 | 7593 | 10545 | 1820 |
|  | 7 | 76,268 | 4374 | 1604 | 114 | 2147 | 3351 | 4166 | 5125 | 7565 | 10545 | 1774 |
|  | 8 | 76,685 | 4369 | 1589 | 114 | 2159 | 3363 | 4154 | 5113 | 7529 | 10545 | 1749 |
|  | 9 | 76,887 | 4332 | 1550 | 114 | 2153 | 3363 | 4143 | 5059 | 7357 | 10545 | 1695 |
|  | Last 31 days | 76,927 | 4325 | 1547 | 114 | 2146 | 3359 | 4137 | 5056 | 7357 | 10545 | 1697 |
| **AMP**  **(N/cm^3^)** | 1 | 71,526 | 882 | 306 | 97 | 437 | 676 | 858 | 1074 | 1441 | 2002 | 398 |
|  | 2 | 71,962 | 874 | 306 | 97 | 425 | 664 | 852 | 1066 | 1436 | 2002 | 402 |
|  | 3 | 72,660 | 871 | 307 | 97 | 425 | 659 | 851 | 1064 | 1431 | 2002 | 405 |
|  | 4 | 73,127 | 867 | 305 | 97 | 423 | 659 | 846 | 1059 | 1428 | 2002 | 400 |
|  | 5 | 73,855 | 865 | 303 | 97 | 425 | 659 | 846 | 1054 | 1420 | 2002 | 395 |
|  | 6 | 74,152 | 864 | 303 | 97 | 425 | 660 | 844 | 1049 | 1431 | 2002 | 389 |
|  | 7 | 74,615 | 863 | 303 | 97 | 429 | 660 | 839 | 1046 | 1431 | 2002 | 386 |
|  | 8 | 75,031 | 863 | 301 | 97 | 428 | 660 | 841 | 1048 | 1423 | 2002 | 388 |
|  | 9 | 75,201 | 861 | 305 | 97 | 427 | 649 | 836 | 1045 | 1432 | 2002 | 396 |
|  | Last 31 days | 75,273 | 860 | 304 | 97 | 425 | 649 | 837 | 1044 | 1431 | 2002 | 395 |
| **BC**  **(µg/m^3^)** | 1 | 79,327 | 0.55 | 0.18 | 0.23 | 0.30 | 0.41 | 0.52 | 0.69 | 0.89 | 1.11 | 0.27 |
|  | 2 | 80,032 | 0.55 | 0.18 | 0.23 | 0.29 | 0.41 | 0.52 | 0.68 | 0.89 | 1.11 | 0.28 |
|  | 3 | 80,566 | 0.55 | 0.18 | 0.23 | 0.29 | 0.40 | 0.52 | 0.68 | 0.89 | 1.11 | 0.28 |
|  | 4 | 81,085 | 0.54 | 0.18 | 0.23 | 0.29 | 0.40 | 0.51 | 0.68 | 0.89 | 1.11 | 0.28 |
|  | 5 | 81,692 | 0.54 | 0.18 | 0.23 | 0.28 | 0.40 | 0.51 | 0.67 | 0.88 | 1.11 | 0.27 |
|  | 6 | 82,267 | 0.54 | 0.18 | 0.23 | 0.28 | 0.39 | 0.51 | 0.67 | 0.88 | 1.11 | 0.28 |
|  | 7 | 82,800 | 0.54 | 0.18 | 0.23 | 0.29 | 0.39 | 0.51 | 0.67 | 0.88 | 1.11 | 0.27 |
|  | 8 | 83,279 | 0.54 | 0.18 | 0.23 | 0.29 | 0.39 | 0.51 | 0.67 | 0.88 | 1.11 | 0.28 |
|  | 9 | 83,895 | 0.54 | 0.18 | 0.23 | 0.29 | 0.39 | 0.51 | 0.67 | 0.88 | 1.11 | 0.28 |
|  | Last 31 days | 83,779 | 0.54 | 0.18 | 0.23 | 0.29 | 0.39 | 0.51 | 0.67 | 0.88 | 1.11 | 0.28 |
| **SO_2_**  **(ppb)** | 1 | 85,085 | 2.38 | 1.56 | 0.34 | 0.56 | 0.94 | 1.98 | 3.68 | 5.11 | 6.76 | 2.74 |
|  | 2 | 85,674 | 2.38 | 1.57 | 0.34 | 0.56 | 0.93 | 1.95 | 3.68 | 5.11 | 6.76 | 2.76 |
|  | 3 | 85,676 | 2.36 | 1.58 | 0.34 | 0.55 | 0.91 | 1.92 | 3.70 | 5.12 | 6.76 | 2.78 |
|  | 4 | 85,507 | 2.35 | 1.58 | 0.34 | 0.55 | 0.90 | 1.89 | 3.69 | 5.14 | 6.76 | 2.80 |
|  | 5 | 85,403 | 2.32 | 1.57 | 0.34 | 0.55 | 0.88 | 1.86 | 3.62 | 5.11 | 6.76 | 2.74 |
|  | 6 | 85,317 | 2.28 | 1.57 | 0.34 | 0.55 | 0.86 | 1.82 | 3.56 | 5.12 | 6.76 | 2.70 |
|  | 7 | 85,436 | 2.24 | 1.55 | 0.29 | 0.53 | 0.84 | 1.78 | 3.44 | 5.10 | 6.76 | 2.60 |
|  | 8 | 85,260 | 2.20 | 1.53 | 0.29 | 0.53 | 0.83 | 1.74 | 3.36 | 5.02 | 6.76 | 2.53 |
|  | 9 | 85,290 | 2.17 | 1.51 | 0.29 | 0.53 | 0.83 | 1.71 | 3.26 | 4.93 | 6.76 | 2.44 |
|  | Last 31 days | 85,276 | 2.16 | 1.51 | 0.29 | 0.53 | 0.82 | 1.70 | 3.25 | 4.93 | 6.76 | 2.42 |
| **O_3_**  **(ppb)** | 1 | 85,234 | 25.8 | 6.7 | 11.7 | 15.4 | 20.1 | 25.9 | 31.7 | 36.0 | 41.0 | 11.6 |
|  | 2 | 85,674 | 25.8 | 6.7 | 11.7 | 15.3 | 20.0 | 25.8 | 31.7 | 36.0 | 41.0 | 11.7 |
|  | 3 | 85,676 | 25.9 | 6.8 | 11.7 | 15.3 | 20.1 | 25.9 | 31.8 | 36.0 | 41.0 | 11.7 |
|  | 4 | 85,507 | 26.0 | 6.8 | 11.7 | 15.4 | 20.1 | 26.3 | 32.1 | 36.0 | 41.0 | 12.0 |
|  | 5 | 85,403 | 26.2 | 6.8 | 11.7 | 15.4 | 20.2 | 26.6 | 32.4 | 36.0 | 41.0 | 12.1 |
|  | 6 | 85,317 | 26.3 | 6.8 | 11.7 | 15.5 | 20.3 | 27.1 | 32.6 | 36.0 | 41.0 | 12.2 |
|  | 7 | 85,436 | 26.5 | 6.7 | 11.7 | 15.8 | 20.6 | 27.3 | 32.6 | 36.2 | 41.0 | 12.0 |
|  | 8 | 85,260 | 26.6 | 6.6 | 11.7 | 16.1 | 20.8 | 27.4 | 32.7 | 36.1 | 41.0 | 11.9 |
|  | 9 | 85,290 | 26.7 | 6.6 | 11.7 | 16.4 | 21.0 | 27.3 | 32.6 | 36.2 | 41.0 | 11.6 |
|  | Last 31 days | 85,276 | 26.7 | 6.6 | 11.7 | 16.4 | 21.0 | 27.3 | 32.7 | 36.2 | 41.0 | 11.6 |
| **Temperature**  **(C◦)** | 1 | 87,094 | 11.0 | 9.4 | -8.9 | -3.3 | 2.0 | 11.5 | 19.9 | 24.0 | 26.3 | 18.0 |
|  | 2 | 87,071 | 10.8 | 9.5 | -8.9 | -3.5 | 1.7 | 10.7 | 19.8 | 24.0 | 26.3 | 18.1 |
|  | 3 | 87,116 | 10.6 | 9.5 | -8.9 | -3.5 | 1.6 | 10.5 | 19.5 | 24.0 | 26.3 | 18.0 |
|  | 4 | 86,961 | 10.5 | 9.5 | -8.9 | -3.6 | 1.6 | 10.4 | 19.6 | 23.9 | 26.3 | 18.0 |
|  | 5 | 86,930 | 10.5 | 9.5 | -8.9 | -3.6 | 1.6 | 10.4 | 19.7 | 24.1 | 26.3 | 18.1 |
|  | 6 | 86,915 | 10.7 | 9.6 | -8.9 | -3.6 | 1.6 | 10.6 | 20.2 | 24.3 | 26.3 | 18.5 |
|  | 7 | 86,970 | 11.0 | 9.6 | -8.9 | -3.4 | 1.7 | 11.1 | 20.5 | 24.5 | 26.3 | 18.8 |
|  | 8 | 86,880 | 11.3 | 9.6 | -8.9 | -3.3 | 1.9 | 11.8 | 20.7 | 24.5 | 26.3 | 18.7 |
|  | 9 | 86,908 | 11.5 | 9.6 | -8.9 | -3.3 | 2.3 | 12.1 | 20.8 | 24.5 | 26.3 | 18.5 |
|  | Last 31 days | 86,910 | 11.5 | 9.6 | -8.9 | -3.3 | 2.3 | 12.2 | 20.8 | 24.5 | 26.3 | 18.5 |
| **Relative humidity**  **(%)** | 1 | 87,094 | 66.3 | 6.4 | 43.4 | 54.6 | 62.3 | 66.9 | 70.6 | 76.1 | 83.7 | 8.4 |
|  | 2 | 87,071 | 66.2 | 6.4 | 43.4 | 54.4 | 62.1 | 66.9 | 70.6 | 75.7 | 83.7 | 8.4 |
|  | 3 | 87,116 | 66.0 | 6.4 | 43.4 | 54.4 | 62.0 | 66.7 | 70.4 | 75.5 | 83.7 | 8.5 |
|  | 4 | 86,961 | 65.8 | 6.4 | 43.4 | 54.0 | 61.7 | 66.6 | 70.3 | 75.1 | 83.3 | 8.6 |
|  | 5 | 86,930 | 65.5 | 6.4 | 43.4 | 53.9 | 61.4 | 66.4 | 70.3 | 74.8 | 83.3 | 8.8 |
|  | 6 | 86,915 | 65.3 | 6.3 | 43.4 | 53.7 | 61.2 | 66.2 | 70.0 | 74.6 | 83.3 | 8.8 |
|  | 7 | 86,970 | 65.1 | 6.2 | 43.4 | 53.7 | 61.2 | 65.9 | 69.8 | 74.3 | 78.3 | 8.7 |
|  | 8 | 86,880 | 65.1 | 6.2 | 43.4 | 53.7 | 61.2 | 65.9 | 69.8 | 74.2 | 78.3 | 8.6 |
|  | 9 | 86,908 | 65.1 | 6.1 | 43.4 | 53.7 | 61.2 | 65.9 | 69.7 | 74.0 | 78.3 | 8.5 |
|  | Last 31 days | 86,910 | 65.1 | 6.1 | 43.4 | 53.7 | 61.2 | 65.9 | 69.6 | 74.0 | 78.3 | 8.5 |

**NOTE:** PM_2.5_: fine particles (aerodynamic diameter <2.5 µm); UFP: ultrafine particles (particles with diameters <100 nm); AMP: accumulation mode particles (particles with diameters 100-470 nm); BC: black carbon (a marker of traffic pollution); SO_2_: sulfur dioxide; O_3_: ozone; IQR: Interquartile range.

**Supplementary Table 2A.** Pearson correlation coefficients for PM_2.5_ across 10 gestational months, and BC across 10 gestational months.

|  | **Gestational Month** | **BC** | | | | | | | | | |
| --- | --- | --- | --- | --- | --- | --- | --- | --- | --- | --- | --- |
|  |  | **1** | **2** | **3** | **4** | **5** | **6** | **7** | **8** | **9** | **last 31 days** |
| **PM_2.5_** | **1** | - | 0.722 | 0.626 | 0.489 | 0.364 | 0.251 | 0.254 | 0.259 | 0.331 | 0.339 |
|  | **2** | 0.503 | - | 0.725 | 0.627 | 0.491 | 0.372 | 0.257 | 0.264 | 0.269 | 0.274 |
|  | **3** | 0.196 | 0.504 | - | 0.725 | 0.630 | 0.498 | 0.381 | 0.269 | 0.275 | 0.273 |
|  | **4** | 0.072 | 0.196 | 0.504 | - | 0.722 | 0.623 | 0.490 | 0.372 | 0.260 | 0.272 |
|  | **5** | 0.150 | 0.065 | 0.193 | 0.503 | - | 0.724 | 0.629 | 0.496 | 0.377 | 0.374 |
|  | **6** | 0.317 | 0.141 | 0.060 | 0.188 | 0.499 | - | 0.726 | 0.632 | 0.496 | 0.498 |
|  | **7** | 0.379 | 0.298 | 0.128 | 0.041 | 0.167 | 0.474 | - | 0.733 | 0.634 | 0.632 |
|  | **8** | 0.276 | 0.379 | 0.304 | 0.123 | 0.031 | 0.163 | 0.444 | - | 0.737 | 0.760 |
|  | **9** | 0.035 | 0.274 | 0.380 | 0.291 | 0.118 | 0.033 | 0.146 | 0.441 | - | 0.930 |
|  | **Last 31 days** | 0.053 | 0.260 | 0.358 | 0.286 | 0.126 | 0.048 | 0.160 | 0.481 | 0.854 | - |

**Supplementary Table 2B**. Pearson correlation coefficients for UFP across 10 gestational months, and AMP across 10 gestational months.

|  | **Gestational Month** | **AMP** | | | | | | | | | |
| --- | --- | --- | --- | --- | --- | --- | --- | --- | --- | --- | --- |
|  |  | **1** | **2** | **3** | **4** | **5** | **6** | **7** | **8** | **9** | **last 31 days** |
| **UFP** | **1** | - | 0.589 | 0.378 | 0.256 | 0.290 | 0.370 | 0.343 | 0.257 | 0.175 | 0.185 |
|  | **2** | 0.748 | - | 0.589 | 0.369 | 0.253 | 0.278 | 0.368 | 0.353 | 0.271 | 0.271 |
|  | **3** | 0.612 | 0.746 | - | 0.590 | 0.372 | 0.247 | 0.286 | 0.380 | 0.355 | 0.349 |
|  | **4** | 0.566 | 0.604 | 0.747 | - | 0.589 | 0.360 | 0.247 | 0.277 | 0.362 | 0.352 |
|  | **5** | 0.632 | 0.569 | 0.606 | 0.745 | - | 0.586 | 0.361 | 0.234 | 0.277 | 0.280 |
|  | **6** | 0.593 | 0.628 | 0.557 | 0.600 | 0.747 | - | 0.587 | 0.357 | 0.231 | 0.237 |
|  | **7** | 0.505 | 0.585 | 0.626 | 0.560 | 0.608 | 0.742 | - | 0.589 | 0.361 | 0.361 |
|  | **8** | 0.487 | 0.495 | 0.581 | 0.617 | 0.560 | 0.603 | 0.740 | - | 0.591 | 0.620 |
|  | **9** | 0.469 | 0.488 | 0.495 | 0.576 | 0.623 | 0.545 | 0.599 | 0.739 | - | 0.909 |
|  | **Last 31 days** | 0.471 | 0.481 | 0.498 | 0.573 | 0.615 | 0.554 | 0.595 | 0.758 | 0.943 | - |

**Supplementary Table 2C.** Pearson correlation coefficients for SO_2_ across 10 gestational months, and O_3_ across 10 gestational months.

|  |  | **O_3_** | | | | | | | | | |
| --- | --- | --- | --- | --- | --- | --- | --- | --- | --- | --- | --- |
|  |  | **1** | **2** | **3** | **4** | **5** | **6** | **7** | **8** | **9** | **last 31 days** |
| **SO_2_** | **1** | - | 0.776 | 0.412 | 0.006 | -0.378 | -0.675 | -0.780 | -0.625 | -0.309 | -0.295 |
|  | **2** | 0.898 | - | 0.776 | 0.414 | 0.013 | -0.373 | -0.671 | -0.784 | -0.624 | -0.606 |
|  | **3** | 0.836 | 0.898 | - | 0.780 | 0.423 | 0.024 | -0.366 | -0.678 | -0.795 | -0.767 |
|  | **4** | 0.819 | 0.838 | 0.899 | - | 0.785 | 0.430 | 0.026 | -0.375 | -0.696 | -0.677 |
|  | **5** | 0.798 | 0.821 | 0.841 | 0.903 | - | 0.785 | 0.429 | 0.024 | -0.390 | -0.383 |
|  | **6** | 0.793 | 0.798 | 0.823 | 0.843 | 0.902 | - | 0.784 | 0.423 | 0.003 | -0.002 |
|  | **7** | 0.793 | 0.790 | 0.798 | 0.822 | 0.841 | 0.899 | - | 0.778 | 0.403 | 0.393 |
|  | **8** | 0.780 | 0.794 | 0.796 | 0.802 | 0.821 | 0.840 | 0.902 | - | 0.768 | 0.754 |
|  | **9** | 0.778 | 0.782 | 0.800 | 0.803 | 0.803 | 0.818 | 0.838 | 0.897 | - | 0.949 |
|  | **Last 31 days** | 0.781 | 0.782 | 0.798 | 0.802 | 0.804 | 0.817 | 0.843 | 0.902 | 0.975 | - |

**Supplementary Table 2D.** Pearson correlation coefficients between ambient gestational month 1 and 2 air pollutant concentrations

|  |  | **Month 2** | | | | | |
| --- | --- | --- | --- | --- | --- | --- | --- |
|  |  | **PM_2.5_** | **BC** | **UFP** | **AMP** | **SO_2_** | **O_3_** |
| **Month 1** | **PM_2.5_** | - | 0.600 | 0.433 | 0.692 | 0.555 | 0.147 |
|  | **BC** | 0.598 | - | 0.457 | 0.621 | 0.552 | -0.178 |
|  | **UFP** | 0.431 | 0.454 | - | 0.672 | 0.722 | -0.139 |
|  | **AMP** | 0.689 | 0.617 | 0.670 | - | 0.505 | 0.115 |
|  | **SO_2_** | 0.553 | 0.545 | 0.719 | 0.502 | - | -0.065 |
|  | **O_3_** | 0.161 | -0.162 | -0.132 | 0.138 | -0.050 | - |

**Supplementary Table 2E.** Pearson correlation coefficients between ambient gestational month 3 and 4 air pollutant concentrations

|  |  | **Month 4** | | | | | |
| --- | --- | --- | --- | --- | --- | --- | --- |
|  |  | **PM_2.5_** | **BC** | **UFP** | **AMP** | **SO_2_** | **O_3_** |
| **Month 3** | **PM_2.5_** | - | 0.608 | 0.439 | 0.690 | 0.565 | 0.099 |
|  | **BC** | 0.606 | - | 0.457 | 0.623 | 0.559 | -0.211 |
|  | **UFP** | 0.437 | 0.459 | - | 0.675 | 0.715 | -0.154 |
|  | **AMP** | 0.696 | 0.628 | 0.675 | - | 0.515 | 0.080 |
|  | **SO_2_** | 0.563 | 0.558 | 0.722 | 0.519 | - | -0.098 |
|  | **O_3_** | 0.123 | -0.194 | -0.145 | 0.093 | -0.079 | - |

**Supplementary Table 2F.** Pearson correlation coefficients between ambient gestational month 5 and 6 air pollutant concentrations

|  |  | **Month 6** | | | | | |
| --- | --- | --- | --- | --- | --- | --- | --- |
|  |  | **PM_2.5_** | **BC** | **UFP** | **AMP** | **SO_2_** | **O_3_** |
| **Month 5** | **PM_2.5_** | - | 0.607 | 0.429 | 0.685 | 0.558 | 0.089 |
|  | **BC** | 0.612 | - | 0.452 | 0.622 | 0.566 | -0.218 |
|  | **UFP** | 0.438 | 0.459 | - | 0.673 | 0.741 | -0.149 |
|  | **AMP** | 0.694 | 0.627 | 0.676 | - | 0.540 | 0.080 |
|  | **SO_2_** | 0.567 | 0.563 | 0.722 | 0.520 | - | -0.125 |
|  | **O_3_** | 0.087 | -0216 | -0.163 | 0.065 | -0.115 | - |

**Supplementary Table 2G.** Pearson correlation coefficients between ambient air pollutant concentrations between gestational month 7 and 8.

|  |  | **Month 8** | | | | | |
| --- | --- | --- | --- | --- | --- | --- | --- |
|  |  | **PM_2.5_** | **BC** | **UFP** | **AMP** | **SO_2_** | **O_3_** |
| **Month 7** | **PM_2.5_** | - | 0.584 | 0.415 | 0.655 | 0.524 | 0.106 |
|  | **BC** | 0.585 | - | 0.449 | 0.628 | 0.570 | -0.200 |
|  | **UFP** | 0.424 | 0.453 | - | 0.660 | 0.743 | -0.138 |
|  | **AMP** | 0.659 | 0.632 | 0.665 | - | 0.541 | 0.100 |
|  | **SO_2_** | 0.523 | 0.567 | 0.747 | 0.544 | - | -0.154 |
|  | **O_3_** | 0.091 | -0.211 | -0.139 | 0.092 | -0.141 | - |

**Supplementary Table 2H.** Pearson correlation coefficients between ambient air pollutant concentrations between gestational month 9 and the last 31 days of pregnancy.

|  |  | **Last 31 days** | | | | | |
| --- | --- | --- | --- | --- | --- | --- | --- |
|  |  | **PM_2.5_** | **BC** | **UFP** | **AMP** | **SO_2_** | **O_3_** |
| **Month 9** | **PM_2.5_** | - | 0.584 | 0.407 | 0.655 | 0.519 | 0.128 |
|  | **BC** | 0.586 | - | 0.445 | 0.631 | 0.571 | -0.192 |
|  | **UFP** | 0.415 | 0.451 | - | 0.663 | 0.766 | -0.089 |
|  | **AMP** | 0.657 | 0.635 | 0.665 | - | 0.546 | 0.129 |
|  | **SO_2_** | 0.523 | 0.573 | 0.771 | 0.550 | - | -0.151 |
|  | **O_3_** | 0.125 | -0.190 | -0.092 | 0.132 | -0.152 | - |

**Supplementary Table 3.** Term birth weight change (g) associated with each interquartile range (IQR) increase in mean pollutant concentrations (other pollutants) during each gestational month, by sex of infants.

| **Gestational month** | **Infant**  **sex** | **BC**  **(IQR = 0.28 µg/m^3^)** | | | **UFP**  **(IQR = 1800 N/cm^3^)** | | | **AMP**  **(IQR = 400 N/cm^3^)** | | | **SO_2_**  **(IQR = 2.65 ppb)** | | | **O_3_**  **(IQR = 12 ppb)** | | |
| --- | --- | --- | --- | --- | --- | --- | --- | --- | --- | --- | --- | --- | --- | --- | --- | --- |
|  |  | **n** | **Birth weight change (g)**  **(95% CI)** | **p-value** | **n** | **Birth weight change (g)**  **(95% CI)** | **p-value** | **n** | **Birth weight change (g)**  **(95% CI)** | **p-value** | **n** | **Birth weight change (g)**  **(95% CI)** | **p-value** | **n** | **Birth weight change (g)**  **(95% CI)** | **p-value** |
| 1 | Male | 34,956 | 11.7  (0.7, 22.7) | 0.480 | 32,307 | 5.1  (-2.8, 13.0) | 0.800 | 31,528 | 7.8  (-1.3, 16.9) | 0.255 | 37,630 | -3.9  (-20.2, 12.4) | 0.402 | 37,692 | -13.8  (-30.3, 2.7) | 0.451 |
|  | Female | 33,993 | 15.1  (4.0, 26.2) |  | 31,405 | 6.0  (-1.9, 13.9) |  | 30,665 | 2.9  (-6.3, 12.1) |  | 36,568 | 0.4  (-15.9, 16.7) |  | 36,634 | -17.8  (-34.4, -1.3) |  |
| 2 | Male | 35,246 | -6.0  (-16.8, 4.9) | 0.203 | 32,572 | -0.6  (-8.7, 7.5) | 0.420 | 31,782 | -6.1  (-15.2, 3.0) | 0.557 | 37,934 | 0.4  (-15.7, 16.4) | 0.432 | 37,934 | 4.0  (-12.5, 20.6) | 0.421 |
|  | Female | 34,344 | 0.1  (-10.8, 11.0) |  | 31,601 | 2.3  (-5.9, 10.5) |  | 30,869 | -3.6  (-12.8, 5.6) |  | 36,817 | 4.4  (-11.7, 20.5) |  | 36,817 | -0.3  (-16.9, 16.3) |  |
| 3 | Male | 35,507 | -4.8  (-15.4, 5.8) | 0.172 | 32,889 | -4.2  (-12.5, 4.0) | 0.038 | 32,113 | -5.8  (-14.8, 3.3) | 0.018 | 37,919 | -7.1  (-23.1, 9.0) | 0.049 | 37,919 | 12.8  (-3.8, 29.3) | 0.307 |
|  | Female | 34,563 | 1.6  (-9.0, 12.3) |  | 31,919 | 3.1  (-5.2, 11.4) |  | 31,187 | 4.3  (-4.8, 13.4) |  | 36,832 | 2.9  (-13.2, 19.0) |  | 36,832 | 7.3  (-9.2, 23.8) |  |
| 4 | Male | 35,756 | -3.0  (-13.4, 7.5) | 0.562 | 33,100 | -3.3  (-11.6, 5.0) | 0.014 | 32,361 | -3.8  (-12.7, 5.2) | 0.061 | 37,859 | -7.8  (-23.9, 8.3) | 0.038 | 37,859 | 12.6  (-3.6, 28.7) | 0.543 |
|  | Female | 34,727 | -0.2  (-10.7, 10.3) |  | 32,042 | 5.4  (-2.9, 13.7) |  | 31,320 | 4.2  (-4.9, 13.3) |  | 36,699 | 2.7  (-13.4, 18.8) |  | 36,699 | 9.3  (-6.9, 25.5) |  |
| 5 | Male | 36,031 | -0.5  (-11.0, 10.0) | 0.260 | 33,303 | -5.6  (-13.7, 2.5) | 0.015 | 32,583 | -0.2  (-9.2, 9.0) | 0.307 | 37,805 | -4.6  (-20.9, 11.7) | 0.109 | 37,805 | -8.0  (-23.6, 7.6) | 0.875 |
|  | Female | 34,992 | 4.8  (-5.7, 15.4) |  | 32,436 | 3.0  (-5.1, 11.2) |  | 31,765 | 4.2  (-5.0, 13.4) |  | 36,620 | 3.6  (-12.7, 19.9) |  | 36,620 | -7.1  (-22.8, 8.5) |  |
| 6 | Male | 36,285 | 4.7  (-5.8, 15.2) | 0.880 | 33,420 | -8.2  (-16.1, -0.3) | 0.687 | 32,728 | 0.9  (-8.0, 9.8) | 0.629 | 37,778 | -0.5  (-16.7, 15.7) | 0.297 | 37,778 | -1.5  (-16.8, 13.8) | 0.374 |
|  | Female | 35,282 | 5.4  (-5.2, 16.0) |  | 32,552 | -6.7  (-14.6, 1.1) |  | 31,835 | -1.1  (-10.0, 7.7) |  | 36,603 | 4.9  (-11.5, 21.2) |  | 36,603 | 3.3  (-12.1, 18.6) |  |
| 7 | Male | 36,557 | -6.1  (-16.7, 4.5) | 0.880 | 33,678 | -6.8  (-14.5, 1.0) | 0.811 | 32,982 | -4.7  (-13.6, 4.2) | 0.805 | 37,804 | -4.8  (-21.4, 11.8) | 0.530 | 37,804 | 16.2  (1.1, 31.3) | 0.714 |
|  | Female | 35,522 | -5.4  (-16.0, 5.2) |  | 32,744 | -5.9  (-13.7, 1.9) |  | 32,014 | -5.8  (-14.6, 3.1) |  | 36,666 | -1.5  (-18.2, 15.2) |  | 36,666 | 18.2  (3.1, 33.4) |  |
| 8 | Male | 36,729 | -1.5  (-12.2, 9.1) | 0.523 | 33,908 | 2.5  (-5.1, 10.1) | 0.282 | 33,210 | -0.4  (-9.2, 8.5) | 0.158 | 37,717 | -3.1  (-20.3, 14.2) | 0.300 | 37,717 | -2.6  (-17.8, 12.5) | 0.512 |
|  | Female | 35,750 | 1.5  (-9.2, 12.2) |  | 32,917 | 6.4  (-1.3, 14.1) |  | 32,199 | 5.7  (-3.2, 14.6) |  | 36,625 | 2.4  (-14.9, 19.6) |  | 36,625 | 0.9  (-14.3, 16.1) |  |
| 9 | Male | 37,021 | -10.2  (-21.0, 0.5) | 0.077 | 33,925 | -1.6  (-9.3, 6.1) | 0.129 | 33,169 | -8.3  (-17.0, 0.5) | 0.008 | 37,689 | -12.6 (-29.6, 4.5) | 0.178 | 37,689 | -3.4  (-18.7, 12.0) | 0.156 |
|  | Female | 35,988 | -2.00  (-12.8, 8.8) |  | 33,047 | 4.0  (-3.8, 11.7) |  | 32,334 | 2.9  (-6.0, 11.7) |  | 36,608 | -5.4  (-22.6, 11.7) |  | 36,608 | 4.5  (-10.9, 19.9) |  |
| Last 31 days | Male | 36,969 | -10.0  (-20.6, 0.6) | 0.079 | 33,951 | -4.9  (-12.4, 2.7) | 0.067 | 33,228 | -9.4  (-18.0, -0.8) | 0.003 | 37,712 | -13.6  (-30.3, 3.1) | 0.324 | 37,712 | -1.4  (-15.1, 12.4) | 0.471 |
|  | Female | 35,916 | -1.8  (-12.4, 8.8) |  | 33,017 | 2.0  (-5.6, 9.5) |  | 32,316 | 3.0  (-5.7, 11.6) |  | 36,569 | -8.4  (-25.2, 8.4) |  | 36,569 | 2.6  (-11.3, 16.5) |  |

**NOTE:** UFP: ultrafine particles (particles with diameters <100 nm); AMP: accumulation mode particles (particles with diameters 100-470 nm); BC: black carbon (a marker of traffic pollution); SO_2_: sulfur dioxide; O_3_: ozone. Models adjusted for temperature of each gestational month, gestational ages, year of birth, month of conception, parity, maternal education, maternal country of birth, maternal race, maternal ethnicity, maternal tobacco use, maternal drug use, maternal pre-pregnancy BMI, previous preterm birth, previous cesarean section, pre-pregnancy diabetes, pre-pregnancy hypertension, hospital of birth, trimester of first prenatal care visit, primary provider of prenatal care, primary payer for prenatal care.

**Supplementary Table 4.** Term birth weight change (g) associated with each IQR increase in mean pollutant concentrations (other pollutants) during each gestational month, by the presence of pregnancy complications.

| **Gestational month** | **Pregnancy complications ^a^** | **BC**  **(IQR = 0.28 µg/m^3^)** | | | **UFP**  **(IQR = 1800 N/cm^3^)** | | | **AMP**  **(IQR = 400 N/cm^3^)** | | | **SO_2_**  **(IQR = 2.65 ppb)** | | | **O_3_**  **(IQR = 12 ppb)** | | |
| --- | --- | --- | --- | --- | --- | --- | --- | --- | --- | --- | --- | --- | --- | --- | --- | --- |
|  |  | **n** | **Birth weight change (g)**  **(95% CI)** | **p-value** | **n** | **Birth weight change (g)**  **(95% CI)** | **p-value** | **n** | **Birth weight change (g)**  **(95% CI)** | **p-value** | **n** | **Birth weight change (g)**  **(95% CI)** | **p-value** | **n** | **Birth weight change (g)**  **(95% CI)** | **p-value** |
| 1 | No | 50,943 | 15.6  (4.5, 26.7) | 0.884 | 47,116 | 7.4  (-14.3, 29.2) | 0.415 | 45,984 | 6.6  (-2.5, 15.6) | 0.929 | 54,795 | -1.4  (-17.1, 14.4) | 0.790 | 54,891 | -17.0  (-33.0, -1.1) | 0.478 |
|  | Yes | 18,006 | 7.4  (-14.3, 29.2) |  | 16,596 | 1.3  (-14.2, 16.8) |  | 16,209 | 2.1  (-15.3, 19.5) |  | 19,403 | -2.9  (-20.6, 14.8) |  | 19,435 | -12.7  (-30.8, 5.4) |  |
| 2 | No | 51,454 | 3.4  (-7.5, 14.2) | 0.148 | 47,401 | -24.1  (-45.9, -2.4) | 0.069 | 46,262 | -2.6  (-11.6, 6.4) | 0.241 | 55,245 | 2.5  (-13.0, 18.0) | 0.927 | 55,245 | 0.2  (-15.8, 16.2) | 0.381 |
|  | Yes | 18,136 | -24.1  (-45.9, -2.4) |  | 16,772 | -11.1  (-27.2, 5.1) |  | 16,389 | -12.7  (-30.6, 5.1) |  | 19,506 | 2.0  (-15.5, 19.4) |  | 19,506 | 5.6  (-12.5, 23.7) |  |
| 3 | No | 51,850 | 2.8  (-7.9, 13.4) | 0.242 | 47,866 | -13.7  (-34.7, 7.3) | 0.755 | 46,729 | -0.8  (-9.7, 8.2) | 0.910 | 55,280 | -1.1  (-16.7, 14.5) | 0.543 | 55,280 | 6.7  (-9.2, 22.7) | 0.051 |
|  | Yes | 18,220 | -13.7  (-34.7, 7.3) |  | 16,942 | 1.2  (-15.3, 17.8) |  | 16,571 | -1.4  (-19.3, 16.4) |  | 19,471 | -4.6  (-22.1, 12.8) |  | 19,471 | 18.6  (0.7, 36.6) |  |
| 4 | No | 52,141 | -2.5  (-12.8, 7.9) | 0.874 | 48,082 | 0.9  (-19.6, 21.4) | 0.340 | 46,988 | 0.3  (-8.6, 9.1) | 0.186 | 55,125 | -2.4  (-18.0, 13.2) | 0.898 | 55,125 | 8.9  (-6.7, 24.5) | 0.185 |
|  | Yes | 18,342 | 0.9  (-19.6, 21.4) |  | 17,060 | 0.0  (-16.5, 16.5) |  | 16,693 | -0.1  (-17.7, 17.5) |  | 19,433 | -3.1  (-20.6, 14.4) |  | 19,433 | 16.9  (-0.7, 34.6) |  |
| 5 | No | 52,518 | 2.7  (-7.8, 13.1) | 0.954 | 48,559 | 0.1  (-20.8, 20.9) | 0.974 | 47,515 | 5.7  (-3.2, 14.7) | 0.885 | 55,034 | -0.2  (-16.0 15.6) | 0.779 | 55,034 | -9.6  (-24.6, 5.5) | 0.166 |
|  | Yes | 18,505 | 0.1  (-20.8, 20.9) |  | 17,180 | -1.2  (-17.5, 15.1) |  | 16,833 | -8.4  (-26.6, 9.9) |  | 19,391 | -1.8  (-19.5, 15.9) |  | 19,391 | -1.2  (-18.4, 16.0) |  |
| 6 | No | 52,882 | 2.6  (-7.9, 13.1) | 0.687 | 48,681 | 13.8  (-6.8, 34.5) | 0.021 | 47,620 | -0.6  (-9.2, 8.1) | 0.532 | 54,967 | 1.0  (-14.7, 16.8) | 0.514 | 54,967 | 0.2  (-14.5, 14.9) | 0.617 |
|  | Yes | 18,685 | 13.8  (-6.8, 34.5) |  | 17,291 | -18.2  (-33.9, -2.5) |  | 16,943 | -0.8  (-18.2, 16.7) |  | 19,414 | 4.8  (-12.9, 22.5) |  | 19,414 | 3.3  (-13.7, 20.2) |  |
| 7 | No | 53,277 | -5.9  (-16.5, 4.7) | 0.894 | 49,039 | -5.7  (-26.6, 15.1) | 0.188 | 47,974 | -7.5  (-16.2, 1.2) | 0.734 | 55,041 | -3.4  (-19.5, 12.8) | 0.937 | 55,041 | 17.0  (2.5, 31.5) | 0.772 |
|  | Yes | 18,802 | -5.7  (-26.6, 15.1) |  | 17,383 | -8.4  (-23.6, 6.9) |  | 17,022 | 1.4  (-15.7, 18.5) |  | 19,429 | -2.9  (-21.0, 15.2) |  | 19,429 | 18.8  (2.0, 35.5) |  |
| 8 | No | 53,604 | 3.8  (-6.9, 14.5) | 0.779 | 49,387 | -10.8  (-31.9, 10.3) | 0.205 | 48,316 | 5.3  (-3.3, 13.9) | 0.187 | 54,978 | -0.0  (-16.8, 16.7) | 0.778 | 54,978 | -1.0  (-15.6, 13.5) | 0.787 |
|  | Yes | 18,875 | -10.8  (-31.9, 10.3) |  | 17,438 | 2.8  (-12.3, 17.9) |  | 17,093 | -6.1  (-23.6, 11.5) |  | 19,364 | -1.7  (-20.3, 17.0) |  | 19,364 | 0.7  (-16.2, 17.6) |  |
| 9 | No | 54,042 | -3.9  (-14.7, 6.9) | 0.923 | 49,573 | -13.9  (-35.4, 7.5) | 0.369 | 48,489 | -4.1  (-12.6, 4.5) | 0.471 | 54,942 | -9.4  (-26.0, 7.1) | 0.834 | 54,942 | 1.9  (-12.8, 16.6) | 0.428 |
|  | Yes | 18,967 | -13.9  (-35.4, 7.5) |  | 17,399 | 11.6  (-3.4, 26.7) |  | 17,014 | 1.3  (-16.1, 18.7) |  | 19,355 | -8.2  (-26.8, 10.4) |  | 19,355 | -3.2  (-20.2, 13.9) |  |
| Last 31 days | No | 53,935 | 15.6  (4.5, 26.7) | 0.793 | 49,536 | 7.4  (-14.3, 29.2) | 0.694 | 48,500 | -4.2  (-12.5, 4.2) | 0.438 | 54,942 | -11.2  (-27.3, 5.0) | 0.967 | 54,942 | 1.5  (-11.6, 14.6) | 0.589 |
|  | Yes | 18,950 | 7.4  (-14.3, 29.2) |  | 17,432 | 5.2  (-9.3, 19.7) |  | 17,044 | -1.3  (-18.2, 15.6) |  | 19,339 | -10.9  (-29.1, 7.3) |  | 19,339 | -1.9  (-17.6, 13.8) |  |

**NOTE:** UFP: ultrafine particles (particles with diameters <100 nm); AMP: accumulation mode particles (particles with diameters 100-470 nm); BC: black carbon (a marker of traffic pollution); SO_2_: sulfur dioxide; O_3_: ozone. Models adjusted for temperature of each gestational month, gestational ages, year of birth, gender of infant, month of conception, parity, maternal education, maternal country of birth, maternal race, maternal ethnicity, maternal tobacco use, maternal drug use, maternal pre-pregnancy BMI, previous preterm birth, previous cesarean section, pre-pregnancy diabetes, pre-pregnancy hypertension, hospital of birth, trimester of first prenatal care visit, primary provider of prenatal care, primary payer for prenatal care.

^a^ pregnancy complications include gestational diabetes, gestational hypertension, eclampsia, abnormal birth conditions and fetus at risk.

**Supplementary Table 5.** Term birth weight change (g) associated with each IQR increase in mean pollutant concentrations (other pollutants) during each gestational month, by maternal employment status during pregnancy.

| **Gestational month** | **Maternal**  **employment** | **BC**  **(IQR = 0.28 µg/m^3^)** | | | **UFP**  **(IQR = 1800 N/cm^3^)** | | | **AMP**  **(IQR = 400 N/cm^3^)** | | | **SO_2_**  **(IQR = 2.65 ppb)** | | | **O_3_**  **(IQR =12 ppb)** | | |
| --- | --- | --- | --- | --- | --- | --- | --- | --- | --- | --- | --- | --- | --- | --- | --- | --- |
|  |  | **n** | **Birth weight change (g)**  **(95% CI)** | **p-value** | **n** | **Birth weight change (g)**  **(95% CI)** | **p-value** | **n** | **Birth weight change (g)**  **(95% CI)** | **p-value** | **n** | **Birth weight change (g)**  **(95% CI)** | **p-value** | **n** | **Birth weight change (g)**  **(95% CI)** | **p-value** |
| 1 | Yes | 44,102 | 14.3  (3.7, 24.9) | 0.598 | 40,643 | 7.2  (-0.4, 14.8) | 0.225 | 39,681 | 9.0  (0.3, 17.7) | 0.024 | 47,391 | 0.1  (-15.9, 16.0) | 0.332 | 47,477 | -17.8  (-33.9,-1.6) | 0.344 |
|  | No | 24,847 | 11.7  (-0.1, 23.5) |  | 23,069 | 2.7  (-5.9, 11.2) |  | 22,512 | -1.2  (-11.1, 8.7) |  | 26,807 | -5.2  (-22.1, 11.7) |  | 26,849 | -12.5  (-29.6, 4.7) |  |
| 2 | Yes | 44,471 | -2.1  (-12.5, 8.4) | 0.637 | 40,980 | 3.0  (-4.8, 10.8) | 0.101 | 39,995 | -3.3  (-12.0, 5.4) | 0.326 | 47,786 | 3.2  (-12.5, 19.0) | 0.611 | 47,786 | 0.3  (-15.9, 16.5) | 0.420 |
|  | No | 25,119 | -4.4  (-16.1, 7.2) |  | 23,193 | -3.1  (-11.9, 5.6) |  | 22,656 | -7.7  (-17.6, 2.2) |  | 26,965 | 0.5  (-16.1, 17.2) |  | 26,965 | 4.8  (-12.4, 22.0) |  |
| 3 | Yes | 44,755 | -0.2  (-10.4, 10.1) | 0.413 | 41,315 | 3.9  (-4.1, 11.8) | 0.001 | 40,308 | 1.8  (-6.9, 10.4) | 0.108 | 47,754 | 0.9  (-14.9, 16.6) | 0.107 | 47,754 | 8.7  (-7.4, 24.9) | 0.541 |
|  | No | 25,315 | -4.2  (-15.6, 7.2) |  | 23,493 | -8.7  (-17.5, 0.2) |  | 22,992 | -5.4  (-15.3, 4.4) |  | 26,997 | -7.7  (-24.4, 9.0) |  | 26,997 | 12.1  (-5.0, 29.3) |  |
| 4 | Yes | 45,021 | -1.7  (-11.7, 8.3) | 0.962 | 41,537 | 2.5  (-5.5, 10.5) | 0.294 | 40,594 | -1.6  (-10.2, 7.1) | 0.333 | 47,606 | -1.0  (-16.8, 14.8) | 0.409 | 47,606 | 9.6  (-6.2, 25.4) | 0.503 |
|  | No | 25,462 | -1.5  (-12.6, 9.7) |  | 23,605 | -1.4  (-10.3, 7.4) |  | 23,087 | 2.7  (-6.9, 12.3) |  | 26,952 | -5.3  (-22.0, 11.3) |  | 26,952 | 13.3  (-3.4, 30.0) |  |
| 5 | Yes | 45,344 | 0.1  (-10.0, 10.2) | 0.267 | 41,942 | 1.0  (-6.8, 8.8) | 0.082 | 41,061 | 1.8  (-7.0, 10.5) | 0.896 | 47,530 | 0.8  (-15.2, 16.8) | 0.463 | 47,530 | -7.0  (-22.2, 8.3) | 0.772 |
|  | No | 25,679 | 5.6  (-5.7, 16.9) |  | 23,797 | -5.4  (-14.1, 3.3) |  | 23,287 | 2.4  (-7.4, 12.2) |  | 26,895 | -3.1  (-19.9, 13.8) |  | 26,895 | -8.6  (-24.8, 7.7) |  |
| 6 | Yes | 45,704 | 3.8  (-6.3, 13.9) | 0.497 | 42,149 | -5.3  (-12.8, 2.3) | 0.107 | 41,260 | 1.5  (-6.9, 10.0) | 0.319 | 47,528 | 5.4  (-10.5, 21.3) | 0.086 | 47,528 | 2.3  (-12.7, 17.3) | 0.512 |
|  | No | 25,863 | 7.2  (-4.1, 18.5) |  | 23,823 | -11.3  (-19.7, -2.8) |  | 23,303 | -2.9  (-12.5, 6.7) |  | 26,853 | -3.8  (-20.7, 13.1) |  | 26,853 | -1.4  (-17.3, 14.6) |  |
| 7 | Yes | 46,019 | -6.7  (-16.8, 3.5) | 0.625 | 42,402 | -3.2  (-10.6, 4.2) | 0.019 | 41,488 | -3.0  (-11.5, 5.4) | 0.173 | 47,566 | -1.2  (-17.5, 15.1) | 0.310 | 47,566 | 18.0  (3.3, 32.8) | 0.721 |
|  | No | 26,060 | -4.2  (-15.6, 7.2) |  | 24,020 | -12.0  (-20.5, -3.6) |  | 23,508 | -9.1  (-18.7, 0.5) |  | 26,904 | -6.7  (-24.0, 10.6) |  | 26,904 | 16.0  (0.3, 31.8) |  |
| 8 | Yes | 46,235 | -0.2  (-10.4, 10.1) | 0.937 | 42,621 | 8.1  (0.8, 15.4) | 0.008 | 41,733 | 5.1  (-3.3, 13.6) | 0.121 | 47,470 | 1.2  (-15.7, 18.1) | 0.430 | 47,470 | 0.3  (-14.5, 15.0) | 0.585 |
|  | No | 26,244 | 0.2  (-11.2, 11.7) |  | 24,204 | -2.0  (-10.2, 6.3) |  | 23,676 | -1.8  (-11.4, 7.8) |  | 26,872 | -3.2  (-21.0, 14.7) |  | 26,872 | -2.9  (-18.7, 13.0) |  |
| 9 | Yes | 46,593 | -4.9  (-15.3, 5.4) | 0.511 | 42,734 | 4.4  (-3.0, 11.7) | 0.022 | 41,808 | -1.9  (-10.3, 6.5) | 0.566 | 47,440 | -5.9  (-22.7, 10.9) | 0.121 | 47,440 | 2.6  (-12.3, 17.6) | 0.331 |
|  | No | 26,416 | -8.1  (-19.6, 3.4) |  | 24,238 | -4.5  (-12.9, 3.8) |  | 23,695 | -4.4  (-13.9, 5.1) |  | 26,857 | -14.5  (-32.2, 3.2) |  | 26,857 | -3.0  (-19.0, 13.0) |  |
| Last 31 days | Yes | 46,527 | -4.4  (-14.6, 5.8) | 0.395 | 41,870 | 2.9  (-4.3, 10.0) | 0.002 | 41,870 | -1.4  (-9.6, 6.8) | 0.225 | 47,465 | -7.8  (-24.2, 8.6) | 0.111 | 47,456 | 4.2  (-9.1, 17.6) | 0.086 |
|  | No | 24,193 | -8.6  (-20.0, 2.8) |  | 23,674 | -9.1  (-17.3, -0.9) |  | 23,674 | -6.7  (-16.1, 2.6) |  | 26,825 | -16.7  (-34.0, 0.7) |  | 26,825 | -5.7  (-20.2, 8.9) |  |

**NOTE:** UFP: ultrafine particles (particles with diameters <100 nm); AMP: accumulation mode particles (particles with diameters 100-470 nm); BC: black carbon (a marker of traffic pollution); SO_2_: sulfur dioxide; O_3_: ozone. Models adjusted for temperature of each gestational month, gestational ages, year of birth, gender of infant, month of conception, parity, maternal education, maternal country of birth, maternal race, maternal ethnicity, maternal tobacco use, maternal drug use, maternal pre-pregnancy BMI, previous preterm birth, previous cesarean section, pre-pregnancy diabetes, pre-pregnancy hypertension, hospital of birth, trimester of first prenatal care visit, primary provider of prenatal care, primary payer for prenatal care.

**Supplementary Table 6.** Term birth weight change (g) associated with each IQR increase in mean pollutant concentrations (other pollutants) during each gestational month, by maternal ethnicity.

| **Gestational month** | **Ethnicity** | **BC**  **(IQR = 0.28 µg/m^3^)** | | | **UFP**  **(IQR = 1800 N/cm^3^)** | | | **AMP**  **(IQR = 400 N/cm^3^)** | | | **SO_2_**  **(IQR = 2.65 ppb)** | | | **O_3_**  **(IQR = 12 ppb)** | | |
| --- | --- | --- | --- | --- | --- | --- | --- | --- | --- | --- | --- | --- | --- | --- | --- | --- |
|  |  | **n** | **Birth weight change (g)**  **(95% CI)** | **p-value** | **n** | **Birth weight change (g)**  **(95% CI)** | **p-value** | **n** | **Birth weight change (g)**  **(95% CI)** | **p-value** | **n** | **Birth weight change (g)**  **(95% CI)** | **p-value** | **n** | **Birth weight change (g)**  **(95% CI)** | **p-value** |
| 1 | Non-  Hispanic | 62,094 | 15.6  (5.5, 25.7) | 0.003 | 57,384 | 7.5  (0.3, 14.7) | 0.001 | 56,036 | 6.9  (-1.3, 15.1) | 0.021 | 66,879 | 1.0  (-14.6, 16.6) | <0.001 | 66,990 | -17.5  (-33.2, -1.7) | 0.044 |
|  | Hispanic | 6,855 | -8.5  (-26.2, 9.2) |  | 6,328 | -12.8  (-25.8, 0.1) |  | 6,157 | -10.1  (-25.5, 5.4) |  | 7,319 | -31.0  (-53.2, -8.8) |  | 7,336 | 0.9  (-21.69, 23.43) |  |
| 2 | Non-  Hispanic | 62,676 | -1.1  (-11.0, 8.9) | 0.014 | 57,733 | 2.9  (-4.5, 10.3) | 0.000 | 56,372 | -4.1  (-12.3, 4.1) | 0.251 | 67,379 | 5.2  (-10.2, 20.5) | <0.001 | 67,379 | 1.6  (-14.2, 17.4) | 0.718 |
|  | Hispanic | 6,914 | -20.8  (-38.2, -3.5) |  | 6,440 | -18.3  (-31.3, -5.3) |  | 6,279 | -12.3  (-27.2, 2.7) |  | 7,372 | -26.9  (-48.9, -5.0) |  | 7,372 | 4.9  (-17.8, 27.6) |  |
| 3 | Non-  Hispanic | 63,139 | 0.1  (-9.6, 9.8) | 0.023 | 58,338 | 1.0  (-6.6, 8.6) | 0.006 | 56,993 | 0.4  (-7.8, 8.5) | 0.069 | 67,413 | 0.7  (-14.7, 16.1) | <0.001 | 67,413 | 9.2  (-6.5, 25.0) | 0.374 |
|  | Hispanic | 6,931 | -18.4  (-35.7, -1.1) |  | 6,470 | -15.8  (-28.9, -2.6) |  | 6,307 | -12.8  (-28.1, 2.4) |  | 7,338 | -33.6  (-55.7, -11.6) |  | 7,338 | 17.3  (-5.1, 39.7) |  |
| 4 | Non-  Hispanic | 63,524 | 0.2  (-9.3, 9.7) | 0.022 | 58,650 | 2.7  (-5.0, 10.3) | 0.007 | 57,346 | 1.3  (-6.8, 9.4) | 0.083 | 67,225 | 1.2  (-14.2, 16.6) | <0.001 | 67,225 | 10.3  (-5.0, 25.7) | 0.462 |
|  | Hispanic | 6,959 | -18.1  (-35.0, -1.1) |  | 6,492 | -13.9  (-27.0, -0.7) |  | 6,335 | -11.0  (-25.8, 3.9) |  | 7,333 | -39.6  (-61.4, -17.8) |  | 7,333 | 16.9  (-5.2, 39.1) |  |
| 5 | Non-  Hispanic | 63,988 | 3.4  (-6.1, 13.0) | 0.081 | 59,180 | 0.4  (-7.0, 7.9) | 0.003 | 57,954 | 3.5  (-4.7, 11.8) | 0.023 | 67,071 | 3.0  (-12.6, 18.6) | <0.001 | 67,071 | -7.5  (-22.3, 7.3) | 0.946 |
|  | Hispanic | 7,035 | -10.7  (-27.8, 6.5) |  | 6,559 | -17.5  (-30.6, -4.4) |  | 6,394 | -12.9  (-28.1, 2.3) |  | 7,354 | -34.3  (-56.3, -12.3) |  | 7,354 | -8.1  (-29.8, 13.6) |  |
| 6 | Non-  Hispanic | 64,474 | 6.4  (-3.2, 15.9) | 0.098 | 59,428 | -5.4  (-12.6, 1.7) | 0.001 | 58,181 | 1.6  (-6.3, 9.6) | 0.010 | 67,059 | 5.2  (-10.3, 20.8) | <0.001 | 67,059 | 1.8  (-12.8, 16.3) | 0.254 |
|  | Hispanic | 7,093 | -7.0  (-24.2, 10.1) |  | 6,544 | -25.9  (-38.8,-12.9) |  | 6,382 | -17.2  (-32.4, -2.1) |  | 7,322 | -33.5  (-55.8, -11.1) |  | 7,322 | -8.6  (-30.3, 13.1) |  |
| 7 | Non-  Hispanic | 64,923 | -4.6  (-14.2, 5.1) | 0.138 | 59,871 | -4.9  (-12.0, 2.1) | 0.016 | 58,603 | -3.6  (-11.5, 4.3) | 0.013 | 67,143 | -0.5  (-16.5, 15.4) | <0.001 | 67,143 | 17.9  (3.7, 32.2) | 0.363 |
|  | Hispanic | 7,156 | -16.5  (-33.6, 0.6) |  | 6,551 | -20.3  (-33.5, -7.0) |  | 6,393 | -21.8  (-36.9, -6.6) |  | 7,327 | -33.6  (-56.4, -10.9) |  | 7,327 | 9.6  (-12.0, 31.3) |  |
| 8 | Non-  Hispanic | 65,300 | 2.3  (-7.4, 12.1) | 0.003 | 60,162 | 6.1  (-0.8, 13.0) | 0.007 | 58,897 | 4.7  (-3.3, 12.6) | 0.004 | 67,003 | 2.6  (-14.0, 19.1) | <0.001 | 67,003 | -0.9  (-15.2, 13.4) | 0.978 |
|  | Hispanic | 7,179 | -22.2  (-39.5, -4.9) |  | 6,663 | -10.5  (-23.4, 2.3) |  | 6,512 | -15.8  (-30.7, -0.9) |  | 7,339 | -31.2  (-54.4, -8.0) |  | 7,339 | -0.7  (-22.5, 21.2) |  |
| 9 | Non-  Hispanic | 65,780 | -3.8  (-13.7, 6.1) | 0.002 | 60,311 | 2.7  (-4.2, 9.7) | 0.009 | 59,000 | -1.8  (-9.7, 6.1) | 0.123 | 66,955 | -6.3  (-22.7, 10.1) | 0.001 | 66,955 | 0.5  (-14.0, 15.0) | 0.951 |
|  | Hispanic | 7,229 | -29.0  (-46.3, -11.7) |  | 6,661 | -14.0  (-27.2, -0.8) |  | 6,503 | -12.8  (-27.7, 2.1) |  | 7,342 | -37.2  (-60.4, -14.0) |  | 7,342 | 1.1  (-20.9, 23.0) |  |
| Last 31 days | Non-  Hispanic | 65,681 | -3.5  (-13.1, 6.2) | 0.001 | 60,279 | 0.9  (-5.8, 7.6) | 0.000 | 59,002 | -1.5  (-9.3, 6.2) | 0.009 | 66,961 | -8.3  (-24.3, 7.7) | 0.001 | 66,961 | 0.9  (-12.0, 13.6) | 0.805 |
|  | Hispanic | 7,204 | -30.7  (-47.9, -13.4) |  | 6,689 | -22.6  (-35.7, -9.6) |  | 6,542 | -20.3  (-35.0, -5.5) |  | 7,320 | -40.6  (-63.7, -17.6) |  | 7,320 | -1.5  (-22.4, 19.3) |  |

**NOTE:** UFP: ultrafine particles (particles with diameters <100 nm); AMP: accumulation mode particles (particles with diameters 100-470 nm); BC: black carbon (a marker of traffic pollution); SO_2_: sulfur dioxide; O_3_: ozone. Models adjusted for temperature of each gestational month, gestational ages, year of birth, month of conception, parity, maternal education, maternal country of birth, maternal race, maternal tobacco use, maternal drug use, maternal pre-pregnancy BMI, previous preterm birth, previous cesarean section, pre-pregnancy diabetes, pre-pregnancy hypertension, hospital of birth, trimester of first prenatal care visit, primary provider of prenatal care, primary payer for prenatal care.

**Supplementary Table 7.** Change in birth weight (g) associated with each IQR increase in mean PM pollutant concentrations for each gestational month, after adjustment for the mean ozone concentration in the same gestational month.

| **Pollutant** | **Black carbon**  **(IQR = 0.28 µg/m^3^)** | | | **Ultrafine particles**  **(IQR = 1800 N/cm^3^)** | | | **Accumulation mode particles**  **(IQR = 400 N/cm^3^)** | | | **SO_2_**  **(IQR = 2.65 ppb)** | | |
| --- | --- | --- | --- | --- | --- | --- | --- | --- | --- | --- | --- | --- |
| **Gestational month** | **n** | **Birth weight change (g)** | **95% CI** | **n** | **Birth weight change (g)** | **95% CI** | **n** | **Birth weight change (g)** | **95% CI** | **n** | **Birth weight change (g)** | **95% CI** |
| 1 | 68,390 | 11.61 | 1.48, 21.73 | 62,497 | 3.99 | -3.36, 11.35 | 60,978 | 5.26 | -2.90, 13.41 | 74,198 | -1.73 | -17.22, 13.75 |
| 2 | 69,059 | -2.03 | -12.07, 8.01 | 63,024 | 0.65 | -6.97, 8.27 | 61,502 | -5.18 | -13.36, 2.99 | 74,751 | 2.36 | -12.91, 17.63 |
| 3 | 69,518 | -0.03 | -9.82, 9.77 | 63,608 | -0.90 | -8.77, 6.98 | 62,100 | -1.54 | -9.70, 6.62 | 74,751 | -2.03 | -17.33, 13.27 |
| 4 | 69,931 | -0.10 | -9.62, 9.43 | 63,925 | 2.57 | -5.30, 10.44 | 62,464 | 0.16 | -7.93, 8.24 | 74,558 | -2.48 | -17.80, 12.83 |
| 5 | 70,382 | 1.10 | -8.44, 10.65 | 64,413 | -1.61 | -9.23, 6.01 | 63,022 | 2.75 | -5.47, 10.97 | 74,425 | -0.89 | -16.41, 14.63 |
| 6 | 70,916 | 5.13 | -4.38, 14.64 | 64,601 | -6.96 | -14.28, 0.37 | 63,192 | 0.09 | -7.86, 8.04 | 74,381 | 2.14 | -13.36, 17.63 |
| 7 | 71,416 | -5.03 | -14.60, 4.55 | 65,086 | -5.87 | -13.04, 1.31 | 63,660 | -6.44 | -14.38, 1.49 | 74,470 | -2.51 | -18.39, 13.38 |
| 8 | 71,803 | 0.02 | -9.64, 9.67 | 65,433 | 4.47 | -2.51, 11.46 | 64,017 | 2.67 | -5.25, 10.59 | 74,342 | -0.41 | -16.89, 16.07 |
| 9 | 72,319 | -6.12 | -15.90, 3.66 | 65,539 | 0.68 | -6.34, 7.69 | 64,070 | -3.01 | -10.88, 4.85 | 74,297 | -9.09 | -25.40, 7.22 |
| Last 31 days | 72,206 | -6.06 | -15.69, 3.57 | 65,538 | -1.91 | -8.65, 4.84 | 64,114 | -3.48 | -11.13, 4.18 | 74,281 | -11.1 | -27.01, 4.82 |

**Note:** Adjusted for temperature of each gestational month, gestational ages, year of birth, month of conception, sex of infant, parity, maternal education, maternal country of birth, maternal race, maternal ethnicity, maternal tobacco use, maternal drug use, maternal pre-pregnancy BMI, previous preterm birth, previous cesarean section, pre-pregnancy diabetes, pre-pregnancy hypertension, hospital of birth, trimester of first prenatal care visit, primary provider of prenatal care, primary payer for prenatal care, and IQR ozone concentration.
